# Synergy between Winter Flounder antimicrobial peptides

**DOI:** 10.1038/s44259-023-00010-7

**Published:** 2023-08-10

**Authors:** Maria Clarke, Charlotte K. Hind, Philip M. Ferguson, Giorgia Manzo, Bhumil Mistry, Bingkun Yue, Janis Romanopulos, Melanie Clifford, Tam T. Bui, Alex F. Drake, Christian D. Lorenz, J. Mark Sutton, A. James Mason

**Affiliations:** 1 Institute of Pharmaceutical Science, School of Cancer & Pharmaceutical Science, King’s College London, Franklin-Wilkins Building, 150 Stamford Street, London, SE1 9NH UK; 2 Technology Development Group, UK Health Security Agency, Research and Evaluation, Porton Down, Salisbury, SP4 0JG UK; 3 Centre for Biomolecular Spectroscopy and Randall Division of Cell and Molecular Biophysics, King’s College London, New Hunt’s House, London, SE1 1UL UK; 4 Department of Physics, King’s College London, London, WC2R 2LS UK

**Keywords:** Antibiotics, Peptides, Computational models, Pharmacodynamics

## Abstract

Some antimicrobial peptides (AMPs) have potent bactericidal activity and are being considered as potential alternatives to classical antibiotics. In response to an infection, such AMPs are often produced in animals alongside other peptides with low or no perceivable antimicrobial activity, whose role is unclear. Here we show that six AMPs from the Winter Flounder (WF) act in synergy against a range of bacterial pathogens and provide mechanistic insights into how this increases the cooperativity of the dose-dependent bactericidal activity and potency that enable therapy. Only two WF AMPs have potent antimicrobial activity when used alone but we find a series of two-way combinations, involving peptides which otherwise have low or no activity, yield potent antimicrobial activity. Weakly active WF AMPs modulate the membrane interactions of the more potent WF AMPs and enable therapy in a model of *Acinetobacter baumannii* burn wound infection. The observed synergy and emergent behaviour may explain the evolutionary benefits of producing a family of related peptides and are attractive properties to consider when developing AMPs towards clinical applications.

## Introduction

In marked contrast to the emergence and spread of resistance to post 1930’s antibiotics, antimicrobial peptides (AMPs) have remained an effective component of the innate immune system throughout evolutionary history. Key differences between most antibiotics in clinical use and AMPs are that the latter are rapidly bactericidal, and their dose dependent bactericidal activity is highly cooperative^1,2^. This desirable pharmacodynamic (PD) property should ensure that a smaller “mutant selection window” exists, minimising the selective pressure associated with an attempted therapeutic response. When resistance to AMPs is evolved experimentally in bacteria, commonly using sub-minimal inhibitory concentration (MIC) conditions, reduced sensitivity can be achieved and there is also a risk of cross-resistance between exogenous AMPs and human defence peptides^3^. However, such adaptation is relatively modest and may be limited by evolutionary constraints^4,5^. Therefore, if their enhanced PD properties do indeed mitigate the risk of resistance to AMPs emerging, their rapid production and/or delivery at supra-MIC concentrations would ensure that their utility endures.

Analogous to the use of combination therapies in the clinic to reduce resistance rates^6^, combinations of dissimilar AMPs have been found to have improved PD properties in vitro^2^. However, the AMPs selected were from different organisms and, notwithstanding our own work with temporin L and temporin B from *Rana temporaria*, it is not yet clear the extent to which AMPs from the same organism can combine to improve both antibacterial potency and the cooperativity of the bactericidal activity^7^.

The Winter Flounder (WF), *Pleuronectes americanus*, produces the AMP pleurocidin^8^, whose potent and broad-spectrum antimicrobial activity may be attributed to its dual abilities of both damaging the bacterial plasma membrane^9,10^, but also crossing it to access intracellular targets^11–13^. The balance of the contribution of the two effects to the bactericidal action may vary according to bacterial species and can be impacted by the nutritional environment and hence bacterial metabolism^14^. Analogues of pleurocidin that have enhanced membrane disruptive activity may be more robust and are sufficiently potent to be effective therapeutics even when delivered systemically in exacting models of bacterial lung infection^14^. Since a pipeline portfolio review, commissioned by the Wellcome Trust^15^, “recommends strong support for funding while monitoring for breakthrough insights regarding systemic therapy” for a tier of approaches that include AMPs the potential for pleurocidin and its analogues to be developed further is clear.

Subsequent to the identification of pleurocidin, further WF AMPs were discovered^16,17^. Antibacterial susceptibility testing of pleurocidin (termed WF2) and five further WF AMPs (sequences given in Table 1) revealed WF1a-1 shared WF2’s broad spectrum potency while WF4 had activity towards Gram-negative bacteria only^17^. The antibacterial activities of WF1, WF1a and WF3 were modest or absent^17^. It is not yet known whether any synergies, in terms of potency or any other parameter, exist between the active or seemingly inactive WF AMPs and hence whether the performance of pleurocidin can be improved or whether other combinations may exceed its potential as a therapeutic. Chequerboard assays are commonly used to identify gains synergistic gains in potency for two-way combinations where growth inhibition with less than half the amount of each component required for inhibition when used alone is commonly taken as defining synergy^18^. Such a methodology is cumbersome and inefficient when applied to three-way or higher order synergism and an elegant method has been proposed where the amount of a given antibiotic required to make a small (1%, 5% or 15%) inhibition impact on growth. When these concentrations are mixed in two-way or higher order combinations, there is room for inhibition that surpasses the expected inhibition from additive contributions to be identified for up to at least five-way combinations^19^. To our knowledge, this method has not yet been applied to AMPs.

**Table 1:** WF AMP sequences and selected physicochemical characteristics.

Peptide	Sequence	aa	Gly	Charge		Δ*G*_woct_ − Δ*G*_wif_	
				pH 8	pH 2	pH 8	pH 2
**WF2**/NRC-04 (Pleurocidin)	G**W**GSFF**KK**AA**H**VG**KH**VG**K**AALT**H**YL-NH_2_	25	4 (16%)	+5	+8	0.391	0.562
**WF4/**NRC-06	G**W**GSIF**K**HG**RH**AA**KH**IG**H**AAVN**H**YL-NH_2_	25	4 (16%)	+4	+9	0.301	0.587
**WF1/**NRC-01	G**K**G**RW**L**ER**IG**K**AGGIIIGGAL**DH**L-NH_2_	24	7 (29%)	+3	+6	0.586	0.410
**WF1a/**NRC-02	**W**L**RR**IG**K**GV**K**IIGGAAL**DH**L-NH_2_	20	4 (20%)	+4	+6	0.454	0.329
**WF1a-1/**NRC-03	G**RRKRKW**L**RR**IG**K**GV**K**IIGGAAL**DH**L-NH_2_	26	5 (19%)	+9	+11	0.659	0.551
**WF3**/NRC-05	FLGALI**K**GAI**H**GG**R**FIHGMIQNHH-NH_2_	24	5 (21%)	+3	+7	0.139	0.377

All peptides were amidated at the C-terminus giving an overall increase in nominal charge of +1. There is 60% identity and 96% similarity between WF2 and WF4 (LALIGN/PLALIGN Waterman–Eggert score: 120; 34.7 bits; *E*(1) < 2.2e−08) and 70 and 85%, respectively, for WF1/WF1a. Average hydrophobicity is given on the whole-residue hydrophobicity octanol-interface scale (Δ*G*_woct_ − Δ*G*_wif_) based on the free energy of transfer from water to palmitoyloleoylphosphatidylcholine and to n-octanol (higher number = more hydrophilic)^45^.

Therefore, in the present study we evaluate whether synergy exists in two-way combinations and evaluate the method of Tekin et al. for identifying higher order synergistic combinations of WF AMPs. We test whether synergy affects the in vitro pharmacodynamics that describes their bactericidal activity and determine its impact on outcomes in a *Galleria mellonella* burn wound *Acinetobacter baumannii* infection model. Having established that synergy between WF AMPs is both prevalent and beneficial, both for in vitro PD and in vivo therapy, we use a combination of steady-state and time-resolved biophysical methods to provide mechanistic insight. In particular, we consider how the membrane interaction of selected WF AMPs is affected by the presence of synergistic partners without the formation of hetero-oligomers and how such interactions might underpin both gains in potency but also higher cooperativity in bactericidal action.

## Results

### Spectrum of antimicrobial activity

We first evaluated the spectrum of antimicrobial activity of the six WF AMPs, using a panel of bacterial isolates that are well characterised and whose susceptibility to other AMPs and membrane active antimicrobials is known (Table 2)^7,14,20–22^. Because of the marine origin of the WF peptides we also included two representative *Vibrio* isolates, *V. parahaemolyticus* and *V. vulnificus*. Consistent with the previous work of Patrzykat et al.^17^, we find that both WF2 (pleurocidin) and WF1a-1 are highly potent antimicrobials, with WF2 displaying the broadest spectrum of activity and WF1a-1 the best Gram-negative activity (MICs 0.25–8 µg/ml), notably for *P. aeruginosa*, (Table 2). To explore this further we prepared D-analogues, to mitigate against proteolytic degradation, and separately tested their activity against an extended panel of *P. aeruginosa* isolates (Supplementary Table 1). The performance of D-WF1a-1 is mostly comparable to that of D-pleurocidin, if not a little better. In contrast with WF2, with which it shares substantial sequence identity (Table 1), the antibacterial activity of WF4 is restricted to Gram-negative bacteria and it is also less active against *Klebsiella pneumoniae* and, as previously^17^, ineffective against *P. aeruginosa*. WF4 does however have notable potency against *A. baumannii* and *Vibrio* spp. which is previously undescribed. Again consistent with previous work^17^, we find WF1, WF1a and WF3 are largely inactive but both *A. baumannii* strains are susceptible to WF1, highlighting again the general susceptibility of this species to AMPs^14,20,21^.

**Table 2:** Antimicrobial activity.

Isolate	WF1	WF1a	WF1a-1	WF2 (pleurocidin)	WF3	WF4
Gram-negative						
* Klebsiella pneumoniae* NCTC 13368	*32*	*128*	4	4–8	*64*	*16*
* Klebsiella pneumoniae* M6	*16*	*128–>128*	4	2–8	*64*	*16–32*
* Acinetobacter baumannii* AYE	4–8	*16–32*	2	2–4	*16*	2-4
* Acinetobacter baumannii* ATCC 17978	4–8	*16–32*	1	2	*8–16*	2-4
* Pseudomonas aeruginosa* PAO1	*128*	*>128*	**2**	8–16	*>128*	*64–128*
* Pseudomonas aeruginosa* NCTC 13437	*128*	*>128*	**4–8**	16	*>128*	*128*
* Pseudomonas aeruginosa* RP73	*>128*	*128*	**8**	32	*>128*	*128*
* Escherichia coli* NCTC 12923	*4–8*	*32*	1–2	1	*8–16*	2–4
* Vibrio parahaemolyticus* NCTC 10903	*16*	*32*	0.25	0.5	*16*	*2*
* Vibrio vulnificus* NCTC 13647	*32*	*32*	2	1	*32*	*4*
Gram-positive						
MS *Staphylococcus aureus* ATCC 9144	*32*	*128*	4–8	2–4	8–32	*16–32*
EMR *Staphylococcus aureus*-15 NCTC 13616	*128*	*>128*	8–16	16	8–16	*>128*
EMR *Staphylococcus aureus*-16 NCTC 13277	*128*	*>128*	8–16	4	*16–32*	*128*
VS *Enterococcus faecalis* NCTC 775	*128*	*128–>128*	*128*	32–64	*64–128*	*128*
VR *Enterococcus faecalis* NCTC 12201	*128–>128*	*>128*	*128*	32	*128*	*128*
VR *Enterococcus faecium* NCTC 12204	*128*	*128*	*16–32*	2–4	*32*	*32–64*

Minimal inhibitory concentrations (µg/ml) are given for peptides tested in Mueller–Hinton broth. Italic or bold values indicate, respectively, a significant (factor of >2) reduction or improvement in potency relative to pleurocidin. Modal values are presented from three independently replicates.

*MS* methicillin sensitive, *EMR* epidemic methicillin resistant, *VS* vancomycin sensitive, *VR* vancomycin resistant.

### Synergy

We used three approaches to explore synergy between WF AMPs. First, we used the method of Tekin et al.^19^ to examine the prevalence of two-way and higher order synergy for the WF AMPs against *K. pneumoniae* NCTC 13368 as net Bliss synergy or emergent synergy (Table 3 and Supplementary Tables 2 and 3). Using this methodology, two-way combinations of WF1a/WF1a-1 (*p* = 0.002), WF1a/WF2, WF1a-1/WF3 and WF2/WF3 (all *p* < 0.0001) produced significantly greater growth inhibition than that predicted from an additive combination of each peptide and are thus considered emergent. For higher order conditions, identification of emergent effects, where the synergy is a result of the entire combination rather than an individual pair within the combination, is dependent on identifying growth inhibition that surpasses that achieved with the corresponding lower order synergistic combinations as well as the expected additive effect. However, the ability to identify such emergent effects is impeded in the assay, as presently configured, by the substantial growth inhibition observed for two-way combinations in which each of five of the six WF AMPs are represented (Table 3). This is true even when the individual components produced no detectable inhibition when used alone and likely reflects the highly cooperative and rapidly bactericidal nature of all the WF AMPs. By a less stringent measure of synergy, thirteen of twenty three-way combinations (Table 3), twelve of fifteen four-way combinations and all six-way and five-way combinations produce a significantly greater than additive inhibitory effect (Supplementary Tables 3 and 4).

**Table 3:** Bliss independence models of two-way and three-way combinations.

Two-way combinations					Three-way combinations				
Condition	% Inhibition	Additive threshold	Bliss net inhibition	*p* value	Condition	% Inhibition	Additive threshold	Bliss net	*p* value
WF1/WF1a	4.26	10.02	−5.76	>0.9999	**WF1/WF1a/WF1a1**	**61.16**	**26.53**	**34.62**	**0.0393**
WF1/WF1a1	39.62	21.98	17.64	0.4673	**WF1/WF1a/WF2**	**98.79**	**16.69**	**82.10**	**<0.0001**
WF1/WF2	36.06	12.14	23.92	0.1006	WF1/WF1a/WF3	23.58	10.02	13.56	0.9892
WF1/WF3	−1.61	5.47	−7.07	0.9995	WF1/WF1a/WF3	2.63	11.18	−8.54	>0.9999
WF1/WF4	21.45	6.63	14.82	0.7274	**WF1/WF1a/WF4**	**65.6**	**28.66**	**36.94**	**0.0212**
**WF1a/WF1a1**	**64.23**	**21.06**	**43.17**	**0.0002**	**WF1/WF1a1/WF2**	**73.56**	**21.98**	**51.58**	**0.0003**
**WF1a/WF2**	**71.21**	**11.23**	**59.98**	**<0.0001**	WF1/WF1a1/WF3	35.09	23.14	11.95	0.9976
WF1a/WF3	4.46	4.55	−0.09	>0.9999	**WF1/WF1a1/WF4**	**87.45**	**12.14**	**75.31**	**<0.0001**
WF1a/WF4	−1.57	5.71	−7.28	0.9993	WF1/WF2/WF3	28.07	13.30	14.77	0.9736
WF1a1/WF2	37.72	23.19	14.53	0.7531	WF1/WF2/WF4	11.34	6.63	4.71	>0.9999
**WF1a1/WF3**	**82.53**	**16.51**	**66.01**	**<0.0001**	**WF1a/WF1a1/WF2**	**99.24**	**27.74**	**71.50**	**<0.0001**
WF1a1/WF4	15.96	17.67	−1.71	>0.9999	**WF1a/WF1a1/WF3**	**92.80**	**21.06**	**71.73**	**<0.0001**
**WF2/WF3**	**97.77**	**6.68**	**91.10**	**<0.0001**	**WF1a/WF1a1/WF4**	**60.11**	**22.22**	**37.88**	**0.0164**
WF2/WF4	12.86	7.83	5.03	>0.9999	**WF1a/WF2/WF3**	**98.70**	**11.23**	**87.48**	**<0.0001**
WF3/WF4	−1.16	1.16	−2.32	>0.9999	**WF1a/WF2/WF4**	**78.73**	**12.39**	**66.34**	**<0.0001**
					**WF1a1/WF2/WF3**	**99.16**	**23.19**	**75.97**	**<0.0001**
					WF1a1/WF2/WF4	36.94	24.35	12.59	0.9955
					**WF1a1/WF3/WF4**	**71.08**	**17.67**	**53.41**	**0.0002**
					**WF2/WF3/WF4**	**99.47**	**7.84**	**−91.64**	**<0.0001**

The percentage inhibition of *K*. *pneumoniae* NCTC 13368 in MHB grown in the presence of two-way or three-way combinations of the six WF AMPs according to the method of Tekin et al.^19^. Subtraction of the additive threshold—inhibition expected from adding the contributions from non-interacting WF AMPs—from the inhibition achieved provides the Bliss net inhibition. Two-Way ANOVA with Šídák’s multiple comparisons test identifies combinations (shown in bold) where the % inhibition significantly surpasses the additive threshold and are synergistic, which for two-way (but not three-way) combinations is emergent. % inhibition and additive thresholds are the average of three independent replicates.

Then we sought to identify possible synergistic binary combinations for three different bacterial species, selecting antibiotic resistant isolates (EMRSA-15 NCTC 13616, *K. pneumoniae* NCTC 13368 and *A. baumannii* AYE) while validating the approach used above. We used a screening method where two WF AMPs were mixed at a ratio corresponding to their MICs and serially diluted as in a standard broth microdilution assay (Table 4). The two methods are generally in agreement, with the screen also identifying synergy between WF1a/WF1a-1, WF1a/WF2 and WF2/WF3 against *K. pneumoniae* NCTC 13368. The screen additionally suggests one further combination (WF3/WF4) with the discrepancy attributable to the slightly different methodologies and different stoichiometries used in the two approaches. Across the screen of the three isolates, strong synergy (FIC < 0.50) is found for nine of fifteen possible two-way WF AMP combinations, but this is dependent on the bacterial isolate tested. Only two combinations produce synergy against all three isolates—the combinations of WF1a with either WF2 or WF1a-1—with other combinations producing synergy against two or only one of the isolates. The combinations of WF2/WF3 and WF3/WF4 both produce synergy against both Gram-negative isolates. Notably, all six of the WF AMPs participate in at least one synergistic combination and, in each of the synergistic two-way combinations found, at least one of the partners has limited or no activity when used alone.

**Table 4:** Prevalence of antimicrobial potency synergy in binary combinations of WF AMPs.

Combination	EMRSA-15 NCTC 13616		*K. pneumoniae* NCTC 13368		*A. baumannii* AYE	
	FIC (mean ± SEM)	MICs (µg/ml)	FIC (mean ± SEM)	MICs (µg/ml)	FIC (mean ± SEM)	MICs (µg/ml)
WF1/WF1a	1.50	128/128	1.00	16/64	0.50	2/8
WF1/WF1a-1	0.50	32/2	1.00	16/2	0.50	2/0.5
**WF1/WF2**	**0.19** ± **0.06**	**8/0.25–16/0.5**	**0.31**	**8/0.25**	0.50	2/0.5
**WF1/WF3**	**0.19** ± **0.06**	**8/0.5–16/1**	0.50	8/16	**0.25**	**1/2**
WF1/WF4	2.00	128/128	0.75	16/4	0.75 ± 0.25	2/1 - 4/2
**WF1a/WF1a-1**	**0.38**	**32/2**	**0.25**	**16/0.5**	**0.25**	**4/0.25**
**WF1a/WF2**	**0.19**	**16/0.5**	**0.13**	**8/0.25**	**0.25**	**4/0.25**
**WF1a/WF3**	**0.19**	**16/1**	1.00	64/32	0.50	8/4
WF1a/WF4	1.50	128/128	1.00	64/8	0.50	8/1
WF1a-1/WF2	1.50 ± 0.5	4/2–8/4	1.00	2/2	1.00	1/1
**WF1a-1/WF3**	1.00	4/4	0.50	1/16	**0.19** ± **0.06**	**0.125/1–0.25/2**
WF1a-1/WF4	2.00	8/128	1.50 ± 0.5	2/8–4/16	1.00	1/2
**WF2/WF3**	1.00	2/4	**0.25**	**0.5/8**	**0.25**	**0.25/2**
**WF2/WF4**	**0.25**	**0.5/16**	1.00	2/8	0.50	0.5/1
**WF3/WF4**	1.00	4/64	**0.25**	**8/2**	**0.19** ± **0.06**	**1/0.25–2/0.5**

FICs and the corresponding combination MICs are shown for the simple binary screen. Combinations with FIC < 0.50, i.e. strong synergy, are shown in bold. FICs are the mean of two repeats +/− the SEM where an SEM exists.

Finally, because in previous work we found that an analogue of pleurocidin with lysine residues replaced with arginine residues and formed of D-amino acids was both robust to serum containing mammalian cell culture media and effective in an in vivo EMRSA-15 NCTC 13616 lung infection model^14^, we used Chequerboard assays to see if the D-enantiomer of WF1a (D-WF1a) acts in synergy with D-pleurocidin and/or its analogue D-pleurocidin-KR (Supplementary Table 4). When tested against either EMRSA-15 NCTC 13616 or *P. aeruginosa* RP73 in RPMI (with 5% FBS) strong synergy (FIC < 0.5) is achieved with both combinations. However, in the analogous experiments in MHB synergy is weakened or lost and the data is consistent with subtle changes in mechanism of action or the ability to interact with targets impacting on synergism.

### Synergy between WF AMPs enhances cooperativity of bactericidal activity

Above, we consider synergism purely in terms of gains in antibacterial potency but other changes in WF AMP behaviour may emerge because of synergy. In work by others, for two-way and three-way combinations of different AMPs from different organisms kappa, a parameter that describes the cooperativity of the dose dependent bactericidal killing rate, determined in vitro, was found to increase on average for three-way combinations^1^. In our own work, again conducted in vitro, we found the cooperativity of the temporin L bactericidal activity against EMRSA-15 NCTC 13616 modestly but significantly increased in the presence of another AMP, temporin B, produced by the same frog^7^. The gain in potency, afforded by synergistic combination of the temporins, is however weak and the cooperativity is lower than that achieved by pleurocidin (WF2) alone in the same conditions^7^.

**Fig. 1:**
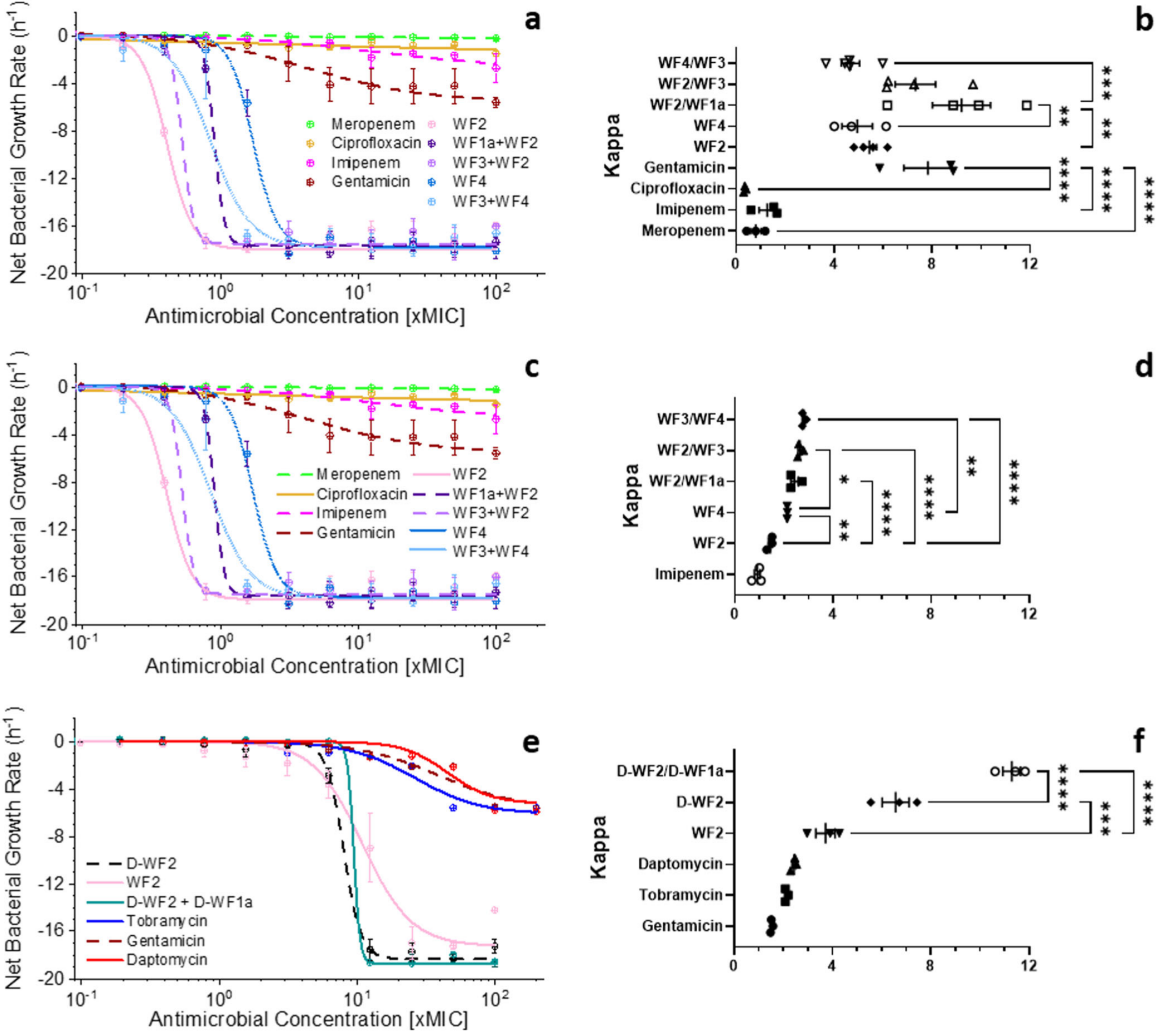
Winter Flounder AMP bactericidal activity is highly cooperative and can be influenced by synergy. In vitro pharmacodynamics assays performed for selected WF peptides and combinations thereof, and bactericidal, clinically relevant antibiotics to which each strain remains susceptible: *A. baumannii* ATCC 17978 in MHB (**a**, **b**), *A. baumanii* AYE in MHB (**c**, **d**) or EMRSA-15 NCTC 13616 in RPMI with 5% FBS (**e**, **f**) were challenged with increasing concentrations of WF AMPs, WF AMP combinations or clinically relevant antibiotics. Curves shown are fits of averages of three independent repeated experiments (**a**, **c**, **e**) and show the change in bactericidal rate as a function of dose, reported as fractions or multiples of the MIC (×MIC). One-way ANOVA with Tukey post hoc test multiple comparisons for Kappa (**b**, **d**, **f**), highlight the differences in cooperativity between the WF AMPs, combinations thereof, and antibiotics. Mean and SE of three independent repeated experiments are shown. Only significant pairwise comparisons between AMPs or between clinically used antibiotics are shown (comparisons between AMPs and antibiotics are provided in the main text): *p < 0.05; **p < 0.01; ***p < 0.001; ****p < 0.0001. Selected time-kill curves, used to construct the PD curves in **a**, are provided in the supplementary material (Supplementary Fig. 1).

In the present study we therefore tested whether the in vitro PD profile of WF2 is affected by its much less potent, synergistic partners WF1a or WF3 and compared this with the action of another WF AMP combination, WF3/WF4, as well as relevant bactericidal antibiotics against three different bacterial isolates in two different conditions (Fig. 1). All WF AMPs and all WF AMP combinations produce substantially faster bactericidal activity than any of the clinically used antibiotics tested, with AMPs killing in minutes and the antibiotics in hours (Supplementary Fig. 1), and this is reflected in the net bacterial growth rates (Fig. 1a, c, e). The maximal killing rate is achieved at or within two times the MIC for ATCC 17978 (Fig. 1a), and approx. ten times the MIC for AYE (Fig. 1c) and EMRSA-15 NCTC 13616 (Fig. 1e). With the exception of gentamicin for *A. baumanii* 17978, which is comparable to all AMPs and AMP combinations, and daptomycin in EMRSA-15 NCTC 13616, which is not inferior to WF2 (but is to D-WF2 and the combination of D-WF2/D-WF1a), this comparison holds also for the cooperativity of the dose dependent activity which is generally much greater for the WF AMPs than what is observed for the clinically used antibiotics (Fig. 1b, d, f).

Since the WF1a and WF3 have relatively weak antibacterial potency, we did not conduct *in vitro* PD experiments for these peptides alone. Therefore, it is moot as to whether the cooperativity of the bactericidal activity for the combinations merely reflects that of the less potent partner or is an emergent property of the combination. Nevertheless, in each of the three separate experiments, the presence of WF1a or its D-enantiomer alongside pleurocidin/WF2 or its D-enantiomer leads to an increase in the cooperativity of the potent and useful bactericidal activity (Fig. 1b, d, f). The presence of WF3 has a similar effect on cooperativity against *A. baumannii* AYE (Fig. 1d) when present with either WF2 or WF4 but this is not observed for ATCC 17978 (Fig. 1b). Further observations include: (1) while the maximal bactericidal killing rate in unaffected, the cooperativity observed for the WF AMPs and combinations is generally lower for the more antibiotic resistant *A. baumannii* AYE (Fig. 1c, d) when compared with the more antibiotic susceptible ATCC 17978 (Fig. 1a, b) and; (2) there is greater cooperativity in the bactericidal action of the D-enantiomer of pleurocidin/WF2 against EMRSA-15 NCTC 13616 (in RPMI with 5% FBS) when compared with that of the L-enantiomer (Fig. 1e, f).

### Synergy between WF AMPs improves therapeutic outcomes

**Fig. 2:**
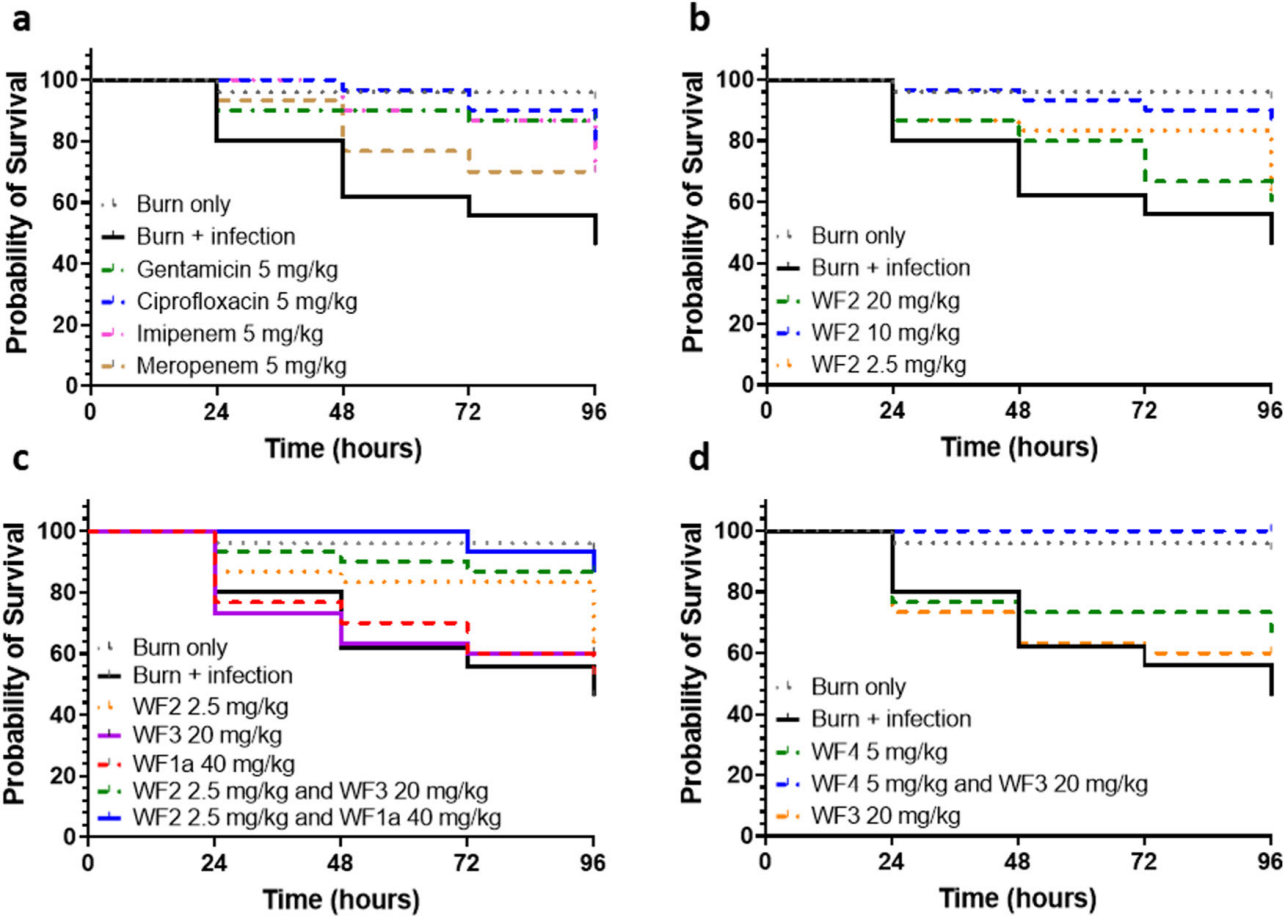
Winter Flounder AMPs are protective when combined in a *Galleria mellonella* model of *Acinetobacter baumannii* ATCC 17978 burn wound infection. Survival curves are plotted for thirty larvae, each treated with classical antibiotics (**a**), WF2 (pleurocidin) (**b**), combinations of WF2 and either WF3 or WF1a (**c**), or WF3 and/or WF4 (**d**), in each case compared with fifty larvae subjected to burn only or burn plus infection (*A. baumannii* ATCC 17978) over 96 h. Percentage survival along with significance tests of protection against infection, due to therapy, according to Log-rank (Mantel–Cox) or Gehan–Breslow–Wilcoxon tests are shown in Table 5.

Having established gains in both potency and cooperativity when combining WF AMPs, we sought to establish whether this translated to gains in therapeutic efficacy in an invertebrate burn wound infection (Fig. 2 and Table 5). Larvae of the greater wax moth, *Galleria mellonella*, have been used recently to establish an invertebrate burn wound infection model^23^. Using this model we have demonstrated therapy with synergistic combinations of gentamicin and bolalipids and established that gentamicin (5 mg/kg), used worldwide for treatment of infected burn wounds, is protective as a monotherapy^22^. Here, gentamicin compares favourably with the other three antibiotic interventions (Fig. 2a and Table 5), and the order of therapeutic success (gentamicin > ciprofloxacin > imipenem > meropenem) is the same as the order of the maximal bactericidal killing rate (Fig. 1a).

**Table 5:** Survival of ATCC 17978 burn wound-infected *Galleria mellonella*.

Condition	Survival % @ 96 h	*p* value	
		Log-rank (Mantel–Cox) test	Gehan–Breslow–Wilcoxon test
Burn only	95.0	<0.0001	<0.0001
Gentamicin 5 mg/kg	**86.7**	**0.0006**	**0.0013**
Ciprofloxacin 5 mg/kg	**80.0**	**0.0017**	**0.0009**
Imipenem 5 mg/kg	**70.0**	**0.0169**	**0.0084**
Meropenem 5 mg/kg	**70.0**	**0.0419**	**0.0488**
WF2 (pleurocidin) 20 mg/kg	60.0	0.2082	0.2633
WF2 (pleurocidin) 10 mg/kg	**86.7**	**0.0004**	**0.0004**
WF2 (pleurocidin) 2.5 mg/kg	63.3	0.0897	0.0813
WF1a 50 mg/kg	53.3	0.5844	0.6668
WF2 2.5 mg/kg/WF1a 50 mg/kg	**86.7**	**0.0002**	**0.0001**
WF3 20 mg/kg	60.0	0.3653	0.6120
WF2 2.5 mg/kg/WF3 20 mg/kg	**86.7**	**0.0005**	**0.0009**
WF4 5 mg/kg	66.7	0.1091	0.1984
WF3 20 mg/kg/WF4 5 mg/kg	**100.0**	**<0.0001**	**<0.0001**

Significance tests are versus burn and infection where survival at 96 h is 46.0%. Conditions where significant (*p* < 0.05) protection is afforded by the treatment are highlighted in bold. Burn only and burn + infection *n* = 50, all treatment groups *n* = 30.

An intermediate dose of 10 mg/kg WF2 (pleurocidin) offers similar protection to that achieved with 5 mg/kg gentamicin but lower (2.5 mg/kg) or higher (20 mg/kg) doses of pleurocidin do not offer significant protection (Fig. 2b and Table 5). In contrast, similar levels of protection to that afforded by gentamicin are achieved with low doses (2.5 mg/kg) of WF2 when combined with either WF1a (50 mg/kg) or WF3 (20 mg/kg) with these two AMPs affording no protection when used alone (Fig. 2c and Table 5). Interestingly a combination of 20 mg/kg WF3 and 5 mg/kg WF4 provides complete protection when use of either of these AMPs alone provides none (Fig. 2d and Table 5).

Having established that synergy between WF AMPs leads to gains in potency, an improved pharmacodynamic profile and therapeutic outcomes we asked whether we could obtain any molecular level insights into how this this might be effected. It is notable that WF2 and WF4 and, separately, WF1 and WF1a share substantial sequence identities and similarities (Table 1) and yet manifestly differ in their antibacterial properties and ability to participate in synergistic combinations. Since a single AMP is unable to form a pore in lipid membrane by itself, some consider the formation of AMP aggregates essential for their activity^24^, and synergism for other AMPs has been explained, at least in part, by the formation of hetero-oligomers^7,25–29^. We therefore conducted a biophysical study of WF AMP conformation and structure as well as their interactions with models of bacterial plasma membranes to characterise and better understand differences in their behaviour and identify aspects that might contribute to synergism.

### WF peptide characteristics

The WF peptides all have the potential to adopt conformations with secondary amphipathicity and are of similar length (Table 1). Of the six peptides, WF3 is noticeably more hydrophobic than the other five peptides, in particular at the N-terminus. WF2, WF3 and WF4 all become more hydrophilic at acidic pH while WF1, WF1a and WF1a-1 conversely become more hydrophobic. All six peptides become more cationic at lower pH. All six peptides are noticeably rich, not only in basic and hydrophobic residues, as is common for cationic amphipathic AMPs, but also in glycine, an amino acid known to confer conformational flexibility.

### WF AMPs are characterised by conformational flexibility and can adopt both α-helix and P_II_ conformations

Previously we have shown that pleurocidin does adopt amphipathic α-helix conformations but is less ordered and/or is characterised by substantial conformational flexibility in models that more accurately reflect the ordered plasma membrane^13,14,30^. Here we present four pieces of evidence that pleurocidin and the other WF peptides can each adopt both α-helix and P_II_ (polyproline-II) conformations.

First, we used far-UV circular dichroism (CD) to study the conformation of all six WF AMPs in aqueous solution as a function of temperature (Supplementary Fig. 2). The spectra obtained at lower temperatures are superficially characteristic of a disordered conformation but on heating there is a substantial change in the spectra of all six AMPs which suggests that at lower temperatures there are contributions to the CD spectra from some ordered or semi-ordered secondary structure. Notably, the intensity of the negative band at 197 nm decreases by almost half while a prominent bump at around 220 nm is also diminished. These features are characteristic of P_II_ conformation even if they are more prominent in model peptides^31^. The spectra obtained at lower temperatures are therefore characteristic of a mixture of disordered and P_II_ conformations, with the latter reduced on heating.

**Fig. 3:**
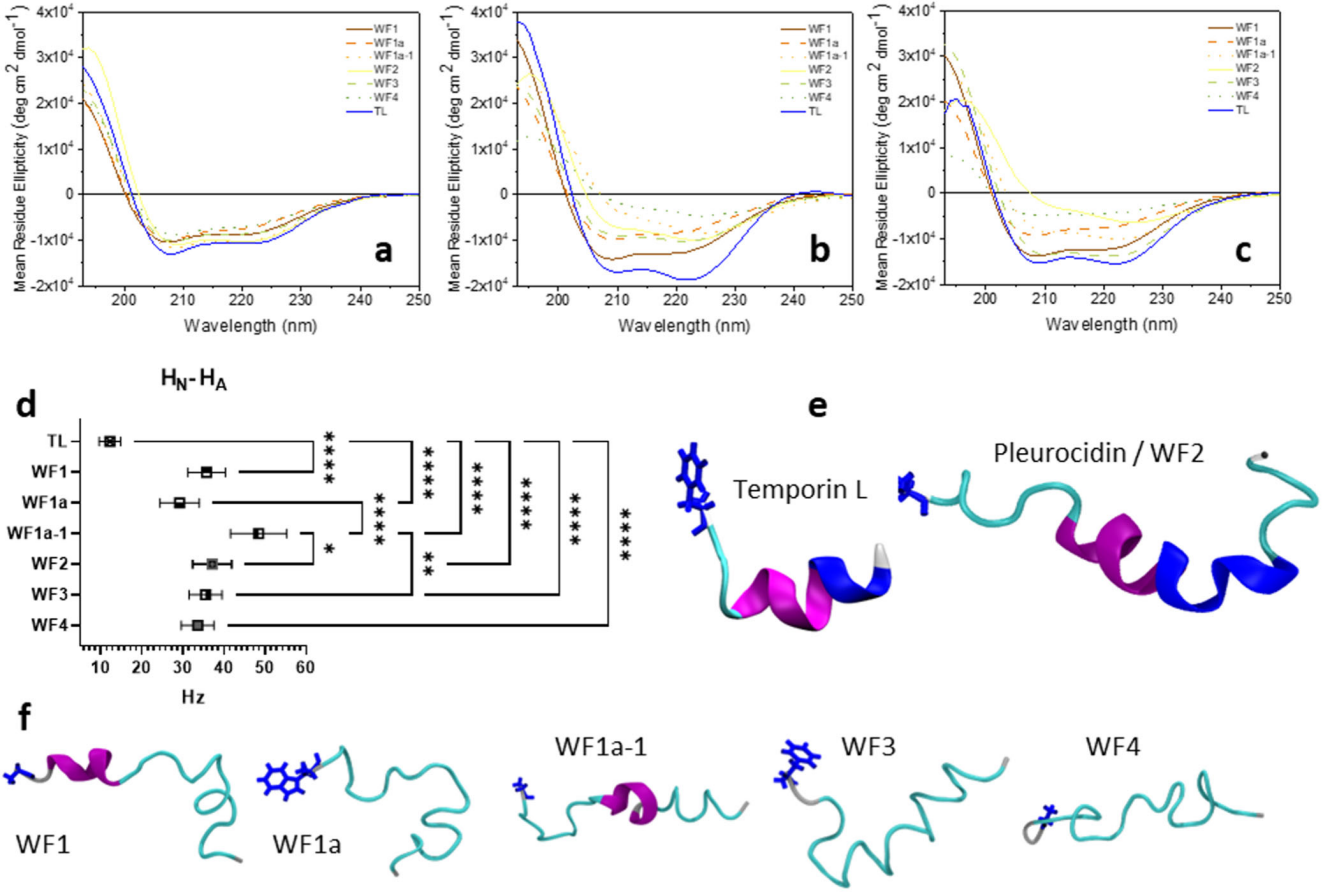
WF peptides do not adopt ideal α-helix conformations in models of bacterial plasma membranes. Far-UV circular dichroism spectra of 50 µM Winter Flounder peptides or temporin L in 5 mM Tris buffer at pH with 5 mM SDS micelles (**a**) or 15 µM in models of Gram-positive (3 mM POPG—**b**) or Gram-negative (3 mM POPE:POPG (75:25)—**c**) bacterial plasma membranes at 37 °C. Spectra are representative of three independently repeated experiments. A comparison of H_N_ to H_A_ NOESY cross peak linewidths (mean and SD) for WF AMPs and temporin L (TL) in SDS_d25_ reveals much greater linewidths for the WF peptides, indicative of intermediate exchange (**d**). Newly solved structures of the WF AMPs are likely hybrids of at least two conformations adopted in SDS (**f**) and lower proportions of α-helix conformation are identified relative to previously solved structures of WF2 (2LS9) and, particularly, temporin L (6GS5) (**e**). Segments are coloured purple for α-helix, blue for 3-_10_ helix and cyan and white respectively for turns and coils. Note however that Define Secondary Structure of Proteins (DSSP) is incapable of identifying P_II_ conformation. One-way ANOVA with Bonferroni’s multiple comparisons test: *p < 0.05; **p < 0.01; ****p < 0.0001.

Second, we used far-UV CD to study the conformation of the WF AMPs in three models of bacterial plasma membranes (Fig. 3a–c). In anionic sodium dodecyl sulfate (SDS) micelles, the CD spectra of all six WF AMPs share characteristics of ordered α-helix conformations with a prominent positive band at 195 nm and negative bands at 208 and 222 nm (Fig. 3a). These however are not as prominent as those observed for temporin L, an AMP with a strong preference for α-helix conformation, and these differences are amplified when the same experiments are performed in lipid bilayers modelling respectively Gram-positive (Fig. 3b) or Gram-negative (Fig. 3c) plasma membranes. The reduction in intensity of these features is consistent both with increased disordering of the α-helix conformation and some adoption of P_II_ conformation with the positive band at 220 nm and negative band at 197 nm expected for P_II_ opposing the spectral features associated with α-helix. Again, the comparison with the CD spectra obtained for temporin L in the same conditions is stark (Fig. 3b, c).

Third, we solved the structures of WF1, WF1a, WF1a-1, WF3 and WF4 in SDS micelles using 2D ^1^H-^1^H NOESY NMR spectroscopy (Fig. 3f). Notably the half-height linewidths for e.g. the H_N_-H_A_ correlations are much larger than expected and significantly so when compared with those obtained for temporin L (Fig. 3d). Such large linewidths are consistent with slow to intermediate exchange on the NMR timescale. In these scenarios the conformationally dependent chemical shifts from two different conformer populations overlap and/or peptides switch from one conformation to another on the same timescale as the NMR experiment so both conformers are sampled. Consistent with this, i-i + 4 NOEs are detectable but weak and do not extend throughout the peptide sequence (Supplementary Fig. 3) and hence, although a coil motif is evident in the resulting structural models, much less α-helix or 3-_10_ helix is detected when compared with previously solved structures of temporin L or even pleurocidin (Fig. 3e).

Finally, we conducted triplicate 200 ns atomistic molecular dynamics (MD) simulations of four of each WF AMP binding and inserting into bilayers comprising either 512 POPG or 384 POPE and 128 POPG lipids modelling, respectively, Gram-positive and Gram-negative plasma membranes. We then assessed the intramolecular H-bonding and, over the last 20 ns of each simulation, average Ramachandran angles for each peptide (Supplementary Fig. 4). Consistent with the NMR structural studies, relatively few intramolecular i-i + 4 (or i-i + 3) hydrogen bonds are detected (Supplementary Fig. 4). Again, although of the WF AMPs WF1 and WF2 have higher numbers of i-i + 4 hydrogen bonds, in POPE/POPG this is dwarfed by the equivalent measure for the strongly α-helical temporin L (Supplementary Fig. 4B). Less intra-molecular hydrogen bonding by WF AMPs relative to temporin L is not offset by increased peptide–lipid hydrogen bonding even if such an effect is observed when replacing lysine residues in WF2 with arginine to create pleurocidin-KR (Supplementary Fig. 4C). Ramachandran plots for WF1a and WF2 (Supplementary Fig. 4D, E) exemplify those observed for the other WFs where individual peptides occupy regions consistent with α-helix (*φ* −60°; *ψ* −45°) and P_II_ (*φ* −75°; *ψ* +150°) conformations. As previously established for pleurocidin^30^, conformational flexibility for the WF AMPs, as determined by the circular variance of *φ* and *ψ* is relatively high, particularly as compared here with e.g. temporin L (Supplementary Fig. 5A, B). However, although conformational flexibility of *φ* for WF1a is reduced when combined with either WF2 or pleurocidin-KR (Supplementary Fig. 5C, D), as exemplified by WF1a and WF2 (Supplementary Fig. 4F), different individual WF peptides are still observed to adopt both conformations in synergistic combinations.

### MD simulations reveal differences in bilayer penetration and lipid acyl chain disordering between WF AMPs

**Fig. 4:**
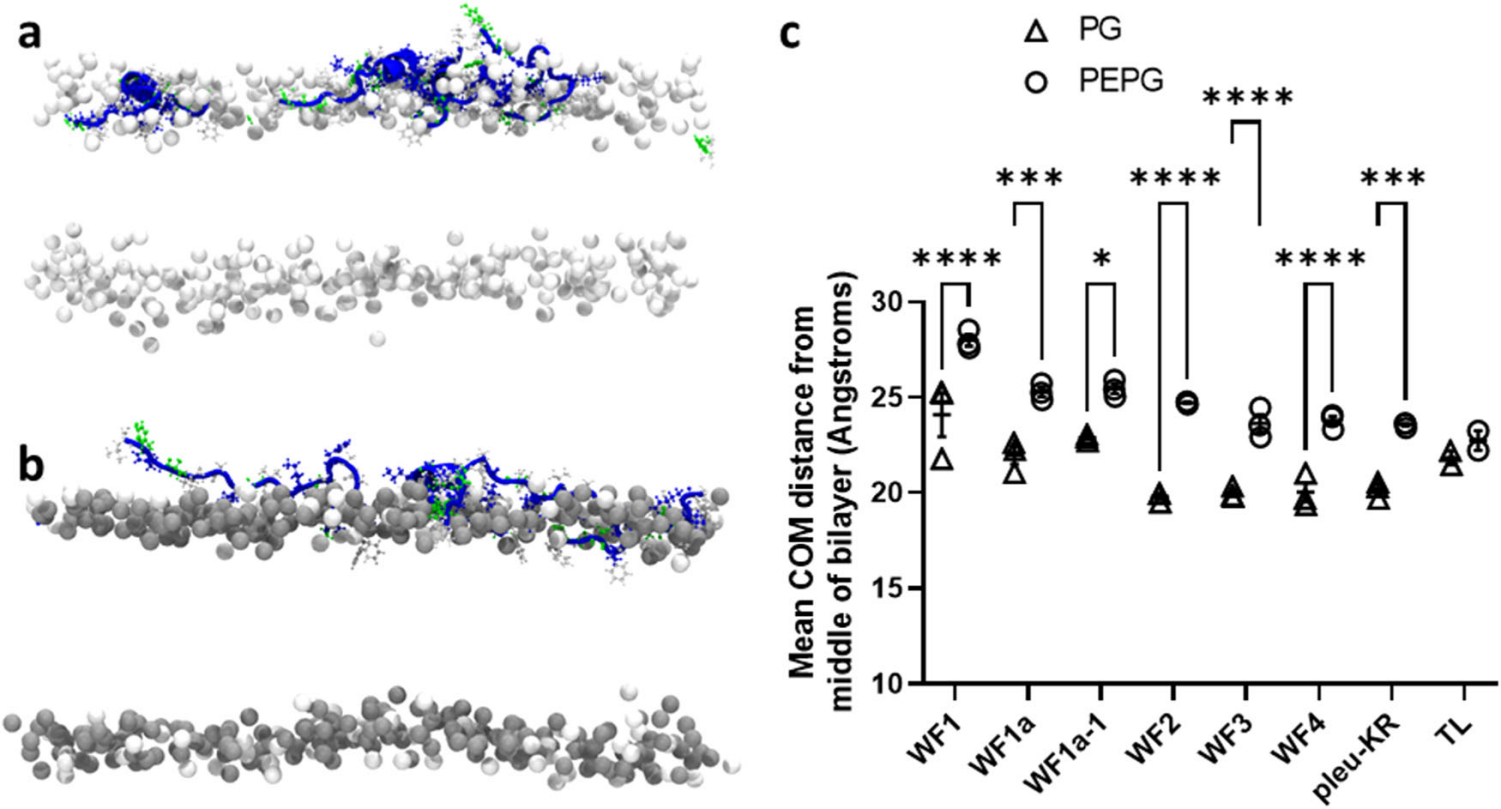
MD simulation—WF peptides penetrate POPG bilayers more readily than POPE/POPG bilayers. Sideview snapshots for WF2/pleurocidin after 200 ns in POPG (**a**) or POPE/POPG (**b**) bilayers—for clarity only the phosphorus atoms are shown for the lipids. Centre of Mass (COM) is shown as averages of the four peptides in each simulation over the last 100 ns in each of three (WF and pleu-KR) or two (temporin L) replicate 200 ns simulations (**c**). Mean and SE shown for replicates. Two-way ANOVA with Šídák’s multiple comparisons test: *p < 0.05; **p < 0.01; ***p < 0.001; ****p < 0.0001.

analysing the MD simulations further we considered whether there are any differences in penetration of the WF AMPs that might explain their different potencies (Fig. 4). Of note, all six WF AMPs and pleurocidin-KR penetrate more readily into the POPG bilayer when compared with the POPE/POPG bilayer, an effect that is not observed for temporin L (Fig. 4c). In POPG WF2 reaches the level of the lipid phosphates (Fig. 4a) and similar penetration is achieved by pleurocidin-KR but also WF3 and WF4, with penetration by WF1, WF1a and WF1a-1 significantly less (Fig. 4c). In POPE/POPG bilayers, WF2 reaches the interfacial region just above the lipid phosphates (Fig. 4b) and this is similar for all peptides bar WF1.

Indeed, of the three WF AMPs that lack antibacterial potency when used alone, WF1 penetrates significantly less into either bilayer than any of the other peptides but WF3 penetrates readily and WF1a penetrates as much as its much more potent analogue, WF1a-1, even if less than WF2 in POPG (Fig. 4c).

To assess the impact on the bilayers of the peptide penetration we calculated lipid acyl chain order parameters for lipids within 4 Å of each inserting peptide. For POPG bilayers, all WF AMPs induce increased disordering (Supplementary Fig. 6A). In the mixed POPE/POPG bilayers, disordering of the anionic PG component due to WF AMPs is similar in the hydrophobic core (between carbons 6 and 14) but WF2 (pleurocidin) disorders the acyl chains more than the other WF AMPs in the region closer to the headgroup (Supplementary Fig. 6B). The disordering effect is generally greater for the PE component and is similar for all WF AMPs except for WF3 which induces greater disorder in the hydrophobic core (Supplementary Fig. 6C).

In previous simulations of eight temporin L peptides binding to the same bilayers as used in the present study we observed long lasting assemblies of three, four or five peptides^7^. Here, with half as many peptides in each simulation the formation of assemblies is less likely though at least one dimer is formed for at least 50 ns by all WF AMPs bar WF3 (Supplementary Figs. 7 and 8). Assemblies are less likely for pleurocidin-KR relative to WF2 but more likely for WF4 and WF1a, with a long-lasting trimer forming in one WF1a simulation (Supplementary Fig. 7). Even though WF3 eschews aggregation when unmixed, in combination a trimer forms and endures in one simulation indicating that hetero-oligomers are possible (Supplementary Fig. 8A). WF AMPs can therefore assemble into dimers, but higher order assemblies are rare at the current peptide surface density.

### Penetration, acyl chain disordering and peptide-lipid hydrogen bonding are altered when WF AMPs are combined

**Fig. 5:**
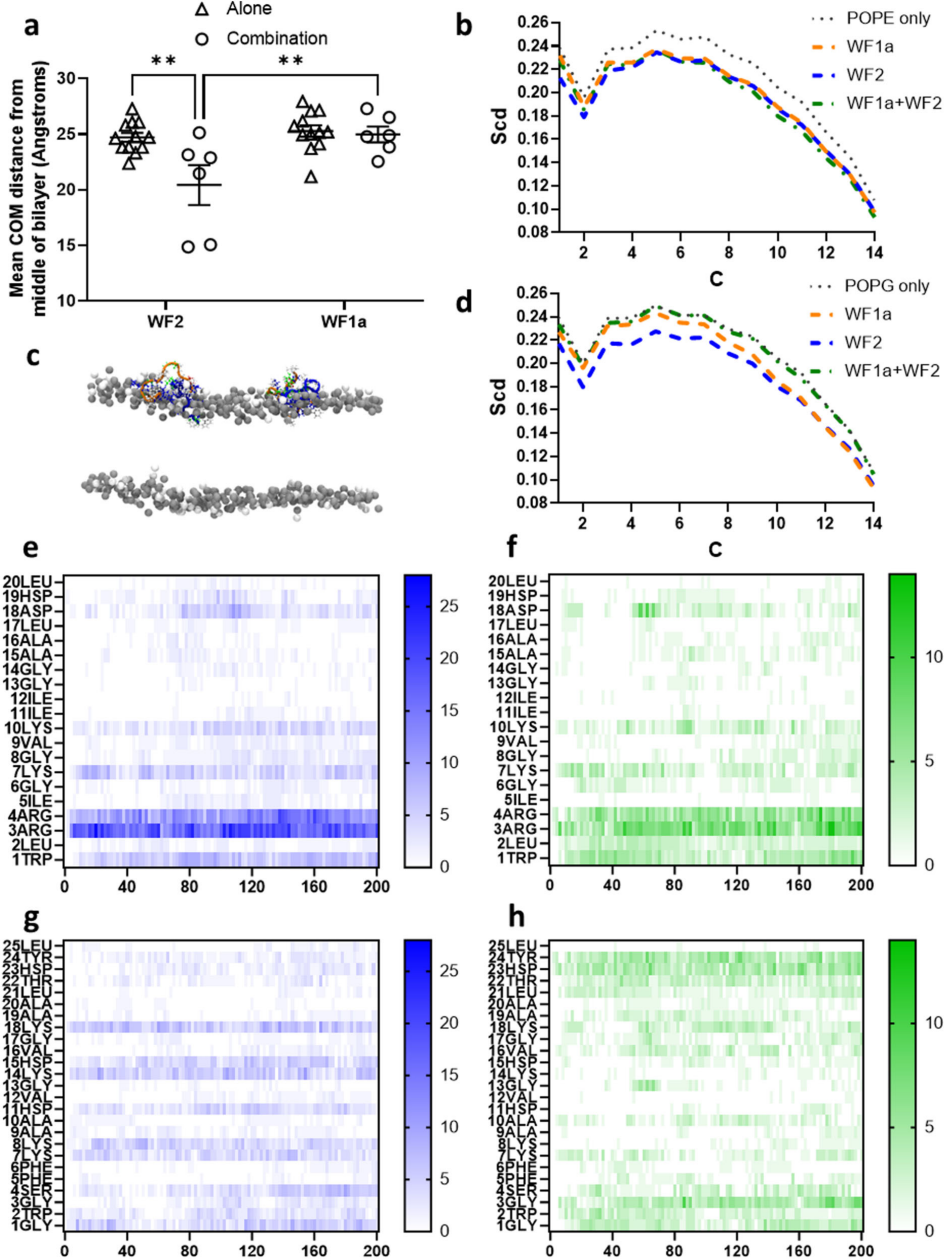
MD simulation—altered penetration and H-bonding distribution in synergistic combinations of W2 and WF1a inserting into POPE/POPG bilayers. Centre of mass analysis where each point is one peptide in one of three replicate simulations, the bar is the mean and error is SE (**a**). Sideview snapshot for WF1a/pleurocidin after 200 ns (**c**). Lipid acyl chain order parameters for lipids within 4 Å of a peptide shown as averages of the three replicates for POPE (**b**) or POPG (**d**) in mixed POPE/POPG bilayers. Hydrogen bonding distribution for WF1a (**e**, **f**) or WF2 (**g**, **h**) run unmixed (**e**, **g**) or as combinations (**f**, **h**). Each panel is a sum of four peptides when the peptides are unmixed or two peptides each for the combinations and is representative of *n* = 3 replicates. Two-way ANOVA with Šídák’s multiple comparisons test: **p < 0.01.

to determine whether any of the properties described above are altered when WF AMPs are present in synergistic combinations, analogous MD simulations were performed where two of each WF AMPs were mixed per the following combinations: WF1a/WF2 in POPE/POPG bilayers (Fig. 5); WF1a/WF2 and WF1a/pleurocidin-KR in POPG bilayers and WF1a/pleurocidin-KR, WF2/WF3 and WF3/WF4 in POPE/POPG bilayers (Supplementary Figs. 9–13).

This maintains the overall number of WF AMPs challenging each bilayer—four—but reduces the number of each type of WF AMP in each simulation. Whereas penetration of WF AMPs, when unmixed, is very consistent between replicate simulations (Fig. 4c), when combined the penetration becomes more variable between replicates and between peptides in each simulation. While some WF2 AMPs penetrate the POPE/POPG bilayer to the same extent as when unmixed, some peptides penetrate much further into the bilayer in the presence of WF1a (Fig. 5a), with part of the peptide penetrating for the first time beyond the plane of the phosphates (Fig. 5b). The same effect is observed for the combination of WF1a and pleurocidin-KR in POPG bilayers (Supplementary Fig. 9A) with similar behaviour observed for WF2 with WF1a in POPG (Supplementary Fig. 9A) and pleurocidin-KR and WF1a in POPE/POPG (Supplementary Fig. 10B) even if there is no significant increase in average penetration. Penetration of POPE/POPG by WF2 is not affected by the presence of WF3 in the same way (Supplementary Fig. 12A) but WF4 does penetrate further when combined with WF3 (Supplementary Fig. 13A), reaching below the lipid phosphate plane (Supplementary Fig. 13C).

For the combinations of WF1a/WF2 and WF2/WF3, disordering of POPG but not POPE lipids is attenuated (Fig. 5b, d and Supplementary Fig. 12B, D), with a similar but less pronounced effect for WF1a/pleurocidin-KR (Supplementary Fig. 11B, D). Disordering induced by the WF3/WF4 combination is broadly similar to that achieved by the unmixed peptides (Supplementary Fig. 13B, D), as is that achieved with the WF1a/WF2 and WF1a/pleurocidin-KR combinations in POPG (Supplementary Figs. 9B and 11A).

To gain insight into the mechanism underpinning these changes in behaviour we considered how the WF AMPs are interacting with the bilayers by quantifying peptide-lipid hydrogen bonding by residue over time (Figs. 5e–h and Supplementary Fig. 9, 10, 12, and 13). While the total number of hydrogen bonds between the peptide and lipids does not change between the unmixed and combination conditions, the pattern observed for WF2 while inserting into POPE/POPG bilayers in the combination is substantially altered while that for WF1a is unaffected (Fig. 5e–h). Notably, strong interactions between lipids and His11 and Lys14 in particular and also Lys8 and His15, that are characteristic of WF2 insertion when unmixed (Fig. 5g), are absent or diminished when in combination with WF1a (Fig. 5h). In contrast, hydrogen bonding at the C-terminus (Thr22, His23, Tyr24) is enhanced (Fig. 5g, h).

The same effect is observed for WF2 inserting into POPG bilayers in the presence/absence of WF1a (Supplementary Fig. 9C–F) and for the analogue pleurocidin-KR inserting into either bilayer, again in the presence of WF1a (Supplementary Fig. 10C–J). For pleurocidin-KR the effect is more dramatic due to the stronger hydrogen bonding potential of its four arginines over the original lysines in WF2/pleurocidin. The presence of WF1a therefore alters the peptide-lipid hydrogen bonding pattern of any peptide that is based on the pleurocidin template. Interestingly the hydrogen bonding pattern of WF2 is also affected by the presence of WF3 (Supplementary Fig. 12E–H) and the same effect is observed for WF4 with which WF2 shares substantial sequence identity and similarity (Supplementary Fig. 13E–H).

In both cases the hydrogen bonding pattern observed for WF3 is unaffected by the presence of either WF2 or WF4, but for both of these peptides the presence of WF3 leads to a substantial enhancement of hydrogen bonding mediated by Gly13/Lys14 (WF2) or Ala13/Lys14 (WF4) at the expense of other interactions which dominate when the peptides are unmixed. As such the MD simulations reveal that less active WF AMPs can modulate the mechanism by which more active WF AMPs interact with lipid bilayers but as yet there is no evidence for the reverse.

### WF AMP synergism is reflected in enhanced membrane activity in ion conductance measurements

Finally we investigated whether the atomic level understanding of the WF AMP membrane interaction afforded by the MD simulations could be related to effects that might be more readily associated with bactericidal activity and the extent to which membrane activity can explain antimicrobial potency and synergy. Indeed, notwithstanding the ability of pleurocidin to penetrate within bacteria to reach intracellular targets^11,12^, it has well-established membrane disruptive properties^9,32^, and both membrane damage and penetration behaviours may be revealed by appropriate ion conductance measurements.

**Fig. 6:**
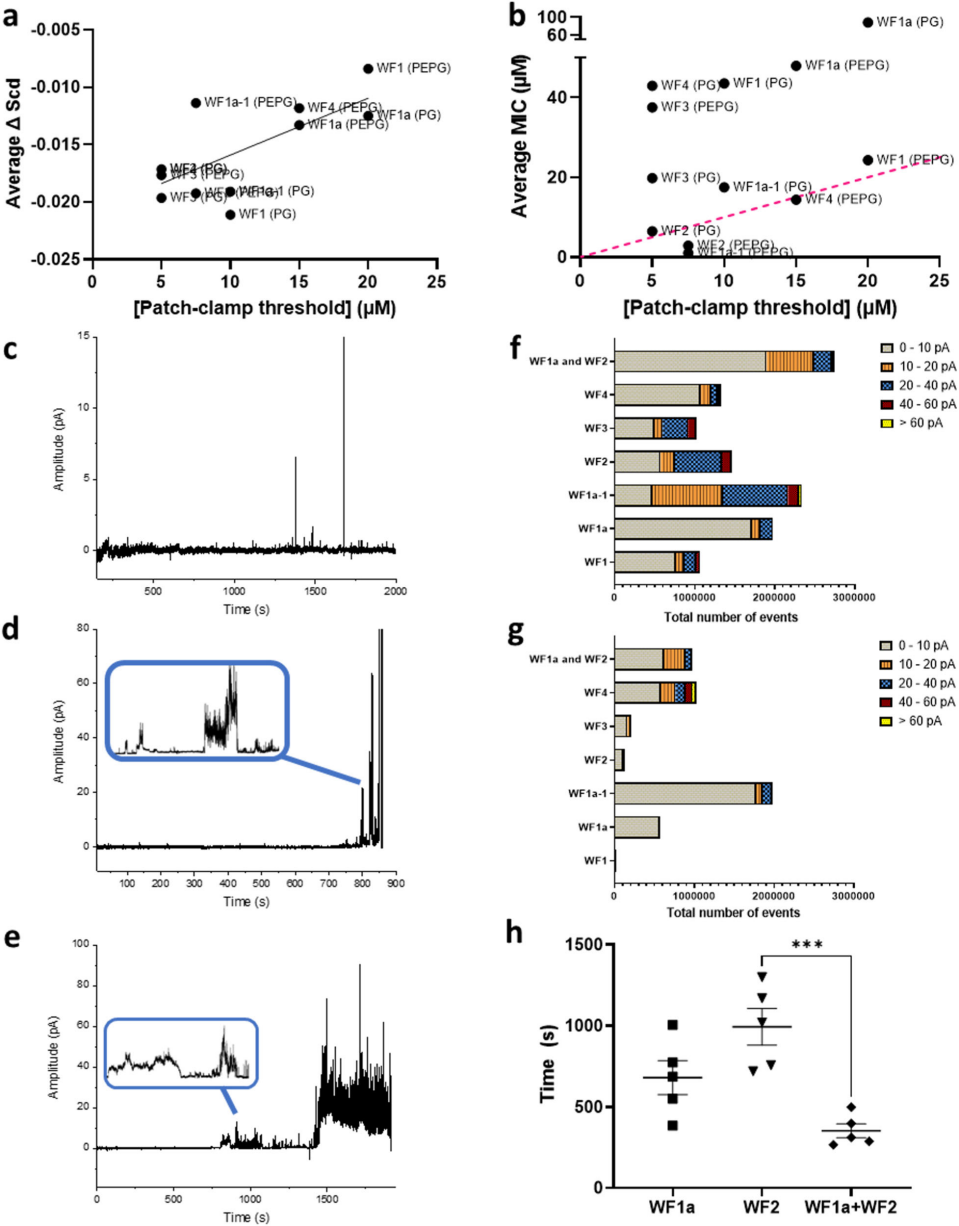
Ion conductance is sensitive to lipid acyl chain disordering induced by WF AMPs and explains synergistic gain in potency. Disordering of lipid acyl chains induced by WF AMPs in MD simulations is related to the lowest concentration at which ion conductance is detected by patch-clamp (**a** Pearson *r* = 0.7007, *p* = 0.0111). At these concentrations, only some WF AMPs trigger substantial membrane activity (**f** DPhPG; **g** DPhPE/DPhPG) and average MICs for Gram-positive or Gram-negative bacteria are unrelated to these threshold concentrations (**b** Pearson *r* = 0.4802; *p* = 0.1141; red dash = line of identity). At its threshold concentration (7.5 µM) WF2, **d**, **g** induces substantial ion conductance in DPhPE/DPhPG bilayers but this is slow (**d**, **h**) and precedes bilayer disruption. WF1a (15 µM) is largely inert (**c**, **g**). Combining WF2 (3.75 µM) and WF1a (5 µM) ensures substantial ion conductance is induced more rapidly and with less peptide (**e**, **g**, **h**). Time taken to onset of conductance (**h**) is the average and SE of five independent replicates. One-way ANOVA with Bonferroni’s multiple comparisons test: ***p < 0.001.

In the patch-clamp experiments our approach has been to titrate peptide to find the lowest threshold concentration capable of triggering ion conductance and then to characterise this membrane activity both qualitatively and quantitatively^7,14,20,21^. Interestingly this threshold concentration, obtained for WF AMPs challenging diphytanoyl lipids, is correlated to the average disordering of the corresponding palmitoyl-oleoyl lipids in the MD simulations (Fig. 6a). A similar but weaker relationship may exist between penetration (COM) and the threshold concentration (Spearman *r* = 0.5593, *p* = 0.0634) but no relationship exists between the threshold concentration and the average MICs for Gram-positive or Gram-negative isolates (Fig. 6b). Therefore, while the ion conductance measurements appear sensitive to variations in the abilities of the WF to penetrate and disorder lipids in model bilayers, such differences do not explain the varying antibacterial potencies.

A better understanding is obtained when the nature of the activity observed at the threshold concentrations is considered as there is substantial variation in the type of activity, the frequency of conductance events, the magnitude of conductance and also the speed with which activity begins after challenge according to the WF AMP applied (Fig. 6c–h and Supplementary Fig. 14). When challenging DPhPG bilayers, modelling Gram-positive bacteria plasma membranes, WF1, WF1a and WF4 have little impact (Fig. 6f) while the onset of activity for WF3 is very slow (Supplementary Fig. 14A). The DPhPE/DPhPG bilayers are generally more resistant to WF AMP induced conductance with only WF1a-1, WF4 and WF2 inducing many high amplitude conductance events (Fig. 6g), while the activity induced by WF2 is slow to begin (Fig. 6d and Supplementary Fig. 14B). Since only these peptides have potent activity against Gram-negative bacteria, these observations are consistent with the relative antibacterial potencies of the WF AMPs described above.

We then focused on whether synergism between WF1a and WF2 can be at all explained by changes in ion conductance. WF1a has very little impact at its threshold concentration (Fig. 6c, g) but when combined with WF2, even at lower concentrations, ion conductance is then substantial (Fig. 6e), with many more high amplitude conductance events (Fig. 6g) and the activity consistently begins much more rapidly (Fig. 6h), the latter being an emergent property since, although this is not significantly faster than when WF1a is used alone (*p* = 0.0658), WF1a alone does not induce conductance that would be associated with bactericidal activity.

## Discussion

In the present study, we show that synergy between AMPs found in the Winter Flounder increases potency, enhances cooperativity in the pharmacodynamics of the bactericidal activity and improves therapeutic outcomes in a model of *A. baumannii* wound infection. We further present a first examination of some of the mechanisms that may underpin the synergy and provide insight into how less active WF AMPs—WF1a or WF3—can modulate the membrane interaction of more active WF AMPs—WF2/pleurocidin or WF4. While successful therapy of infected burn wounds in an invertebrate infection model raises the prospect of translating this to mammalian models and, ultimately the clinic, there remain more fundamental questions. These include whether WF AMPs can participate in emergent synergy in higher order combinations and the extent to which variations in environment, bacterial species and strain might affect cooperativity in bactericidal activity and whether this does indeed affect risk and/or frequency of resistance.

The identification of numerous two-way synergistic combinations of WF AMPs can be achieved using both the elegant and efficient approach of Tekin et al. and a simple broth microdilution of WF AMPs paired according to their individual MICs. In contrast with Tekin *et al*., we are unable to identify emergent higher order synergistic combinations, but this may reflect the rapidly bactericidal and high cooperativity of AMP activity. The approach is dependent on identifying antibiotic doses that lead to small but measurable reductions in growth such that even with higher-order combinations, emergent synergism can be identified provided lower-order combinations do not generate complete inhibition^19^. However, because of the high cooperativity of AMPs, it is possible for a drastic change in bactericidal activity to be achieved with only a small change in AMP concentration. Indeed, it is possible for zero inhibition to be observed with individual WF AMPs and for near total inhibition to be found with only two-way combinations and we show here that cooperativity is enhanced even with two-way combinations. As such we will likely need a different approach to identify higher order synergy between AMPs with the ideal method both being high throughput and capable of providing dose-dependent and time-resolved data to account for any emergent changes in bactericidal killing rate.

In the interim however MD simulations suggest three-way combinations that should be investigated further. Notably, the MD simulations reveal the peptide-lipid hydrogen bonding patterns of both WF2/pleurocidin and WF4 are similarly affected by the presence of WF3 and hence the question arises as to whether WF3 can affect both peptides simultaneously and what the outcome will be of having these two effects in parallel. In contrast, the effects of WF1a and WF3 on the peptide–lipid hydrogen-bonding pattern of WF2/pleurocidin differ substantially. As such the question arises whether these effects might compete, or whether some other behaviour emerges. Notably both WF2 and WF3 are expressed in the gills of the Winter Flounder (along with WF1)^33^, and WF2, WF3 and WF1a are expressed in the intestine and this raises the question as to how environment and/or differences in bacterial threat shape the function of different synergistic combinations. Expression of WF4 was not found in the adult skin or intestine but other tissues and life stages were not sampled and there was no pathogen insult that might trigger expression^33^. The physiological role of WF4 and the combinations in which it might participate therefore remain elusive for now. Nevertheless, it appears likely that co-expression of WF2 with WF1a and/or WF3 in situ can afford gains for the Winter Flounder innate immune system in terms of antibacterial potency which may translate to greater selectivity and reduced collateral damage at the site of infection. Further, while it is yet to be tested, according to the theory advanced by Rolff and others^1,2,34^, the increase in cooperativity of the bactericidal action obtained by combining different WF AMPs may limit the development of resistance to the Winter Flounder’s innate immune response.

In the present study, we are able to establish that poorly active WF AMPs such as WF3 or WF1a are able to modulate the membrane interaction of more active WF AMPs such as WF4 or WF2/pleurocidin. We further show that only those WF AMPs that induce substantial membrane activity at their threshold concentrations have potent antibacterial activity and that this activity is potentiated and accelerated at lower doses of WF2/pleurocidin by WF1a. This effect likely explains the synergistic gain in potency observed in this combination and yet the Gram-negative MICs for both WF2 and WF1a-1 are both well below the threshold concentration in patch-clamp. This would indicate that there is more to the bactericidal mechanism than membrane disruption for both peptides and also that for some bacterial species membrane disruption is a less efficient bactericidal strategy. Therefore, since the ability of WF2/pleurocidin to penetrate bacteria and disrupt intracellular targets is well established for WF2/pleurocidin^11–13^, and no similar study exists for WF1a-1, there is a need to probe non-membrane related contributions to potency and synergy in more depth to fully understand the effect.

While WF AMP effects beyond the bacterial plasma membrane may also influence the cooperativity of bactericidal action—indeed the high cooperativity of gentamicin observed here is consistent with high cooperativity of its binding to two of three classes of binding site on the 70 S ribosome^35^—the ability of MD simulations to identify modulation of WF4 and WF2/pleurocidin binding and insertion in models of the plasma membrane, by WF1a and/or WF3 suggests that this technique may reveal at least part of how the bactericidal pharmacodynamics are altered in two-way combinations. Enhanced cooperativity in bactericidal activity in two-way synergistic combinations requires a relative inhibition of activity at low AMP concentrations and enhancement of activity at higher concentrations. Features that could support each of these effects are observed at the single concentration tested here, notably the perturbation of the peptide-lipid hydrogen bonding pattern observed for WF2/pleurocidin and WF4 in unmixed condition but also the increased penetration of WF2 (with WF1a) and WF4 (with WF3). Dose dependent changes in this behaviour may therefore provide a mechanistic understanding of the importance of these effects and may be combined with dose dependent, time resolved studies of plasma membrane depolarisation and intracellular target disruption to gain a comprehensive understanding of cooperativity and speed of bactericidal action.

We also show here that the ability to adopt both α-helix and polyproline-II conformations in membrane environments is a distinctive feature of WF AMPs. However, its importance is not yet understood as, although the WF AMPs may penetrate phosphatidylglycerol rich bilayers more readily than other AMPs that adopt only α-helix conformations, we have yet to establish any particular benefit of the P_II_ conformation or the ability to swap between the two conformations or any change in this behaviour in WF AMP combinations (beyond a modest reduction in flexibility for WF1a).

In comparing the cooperativity of WF AMP bactericidal activity between bacterial species and between different strains of *A. baumannii*, we can show that the antibacterial pharmacodynamics of AMPs will be strongly influenced by the nature of the bacterial target. The pharmacodynamics of bactericidal action can both improve or deteriorate following antibiotic adaptation^36^, and origin of the lower cooperativity observed here for WF AMPs against the more antibiotic resistant AYE strain, relative to *A. baumannii* ACTCC 19778, warrants further investigation.

In summary, we find that synergy can enhance the potency and bactericidal pharmacodynamics of AMPs from the same organism and expressed in the same tissue and this enhances therapy. The PD profile may however vary according to species and/or strain and there is a need to identify contributions both from bacteria and their environment and the AMPs themselves to understand how PD is manipulated. With such an understanding an investigation of whether and how a varying PD profile is indeed linked to resistance risk can follow and the benefits fully appreciated.

## Methods

### Peptides and lipids

All peptides were purchased from Cambridge Research Biochemicals (Cleveland, UK) as desalted grade (crude). The crude peptides were further purified using water/acetonitrile gradients using a Waters SymmetryPrep C8, 7 µm, 19 × 300 mm column. All peptides were amidated at the C-terminus. The lipids 1-palmitoyl-2-oleoyl-*sn*-glycero-3-phospho-(1′-*rac*-glycerol) (POPG), 1-palmitoyl-2-oleoyl-*sn*-glycero-3-phosphoethanolamine (POPE), 1,2-diphytanoyl-*sn*-glycero-3-phospho-(1'-*rac*-glycerol) (DPhPG) and 1,2-diphytanoyl-*sn*-glycero-3-phosphoethanolamine (DPhPE) were purchased from Avanti Polar Lipids, Inc. (Alabaster, AL) and used without any purification. All other reagents were used as analytical grade or better. Bacterial isolates are from a collection maintained by UKHSA^14^.

### Antibacterial activity assay

The antibacterial activity of the peptides was assessed through a modified two-fold broth microdilution assay with modal MICs generated from at least three biological replicate experiments^37^. The method broadly followed EUCAST methodology, with non-cation adjusted Mueller Hinton replacing cation-adjusted Mueller Hinton. Peptides and antibiotics were diluted in a two-fold dilution in media down a 96 well plate. Bacteria were then added, back-diluted from an overnight culture, at a starting concentration of 5 × 10^5^ CFU/ml. Plates were incubated, static at 37 °C, for 20 h and the OD_600_ was determined using a Clariostar plate reader (BMG Labtech). The MIC was defined as the lowest concentration where growth was <0.1 above the background absorbance.

### High order synergy screen

The method of Tekin et al. was followed under the same conditions are the MICs^19^. Briefly, an antibacterial activity assay was performed in triplicate as above, and the endpoint growth was used to calculate the peptide concentration which inhibited 10% of growth. Peptides at these concentrations were added to wells of a 96-well plate both alone and in combinations of 2, 3, 4, 5 or all 6 peptides to an accumulative total of 100 µl. 100 µl of bacteria at a starting concentration of 5 × 10^5^ CFU/ml were then added and the plate was incubated static at 37 °C for 20 h. The OD_600_ was determined using a Clariostar plate reader (BMG Labtech). The percentage growth inhibition was calculated for each combination compared to an untreated control. Bliss net independence synergy was calculated according to Tekin et al. by subtracting the additive inhibition of the individual peptides from the total inhibition of the combination.

### Synergy screen against three isolates

Pair-wise combinations of peptides were tested following an adjusted MIC method as follows. Combinations of 2 peptides, with each compound at its MIC concentration, were added to the first row of a 96-well plate and diluted down the plate in a two-fold serial dilution. Bacteria were added as in a standard MIC assay, the plate was incubated under the same conditions and the MIC of the combination was defined in the same way. The fractional inhibitory concentration was calculated from the combination MIC for two independent repeats, and presented as the average +/− standard error of the mean. FIC is calculated as (MIC of compound A in combination with B/MIC of compound A alone) + (MIC of compound B in combination with A/MIC of compound B alone). Where the MIC was >128 µg/ml, an assumed MIC of 256 µg/ml was used to calculate the FIC and a top concentration of 128 µg/ml was added in the combination. FIC values ≤0.5 were considered strongly synergistic and, consistent with a recent re-evaluation of FIC which stresses the importance of also measuring the MIC in the same microarray plate, values of 0.5–<1 were weakly synergistic^18^.

### Chequerboard assays

Synergy was measured using standard microdilution Chequerboard assays under the same conditions as the MICs with RPMI140 + 5% FBS used alongside MHB^18^. Twofold dilution series of each peptide or antibiotic were prepared in separate 96 well plates and then combined into one before addition of bacteria. The growth/no growth interface was determined using the same definition as the MIC. The fractional inhibitory concentration was calculated from the most synergistic well on the plate for three independent repeats, and presented as above. MICs were determined on the same plates as the FICs to increase reproducibility.

### In vitro pharmacodynamic assay

In vitro pharmacodynamic assays were performed with epidemic methicillin resistant *S. aureus 15* (EMRSA-15 NCTC 13616) cultured in RPMI140 + 5% FBS or in Mueller-Hinton broth, and *A. baumannii* ATCC 17978 or AYE cultured in Mueller Hinton Broth (MHB). Cation adjusted MHB (CA-MHB) was used when testing daptomycin due to its requirement for Ca^2+^ ions for activity. Bacteria were cultured overnight in 10 ml of MHB or RPMI + 5% FBS at 37 °C and diluted just prior to plate inoculation to an OD_600_ of 0.002. Stock solutions of WF1a, WF2, WF3, WF4, D-WF1a, D-WF2, tobramycin, gentamicin, daptomycin, imipenem, meropenem, or ciprofloxacin were prepared in sterile Milli-Q water at a concentration of 200× MIC. Daptomycin was prepared in methanol at a concentration of 2000× MIC and diluted with media to 200× MIC in the first well. A dilution series was made in the top row of a polypropylene 96-well plate from 200× or 100× MIC (see Supplementary Table 5 for top concentrations for each condition) to 0.2× MIC in a volume of 100 μl, to which 100 μl of the bacterial suspension was added to have a total of 1 × 10^6^ log-phase colony forming units (CFU) in 200 μl. The first *t* = 0 sample was taken <30 s after addition of bacteria to the plate with further samples taken at appropriate intervals thereafter. Peptide-challenged bacteria were sampled every 20 min for 120 min due to rapid killing while tobramycin, gentamicin, daptomycin, imipenem, meropenem and ciprofloxacin challenged bacteria were sampled every hour for 6 h. A volume of 15 μl was removed from each well and, following the drop plate method for enumerating bacteria^38^, diluted 1:1000 in phosphate buffered saline and plated onto MH agar or CA-MH agar plates. The plates were incubated at 37 °C overnight for CFU counting. CFU data were log_10_ transformed, and the bacterial net growth rate was determined from the increase or decrease in CFU during the time of exposure to the peptides or antibiotics as the coefficient of a linear regression of log_10_ CFU as a function of time. The intercept of the regression was fixed by forcing the regression lines through the first CFU measurement (0 min) at a given antimicrobial concentration. The pharmacodynamic function according to Regoes et al. describes the relationship between bacterial net growth rate *ψ* and the concentration of an antimicrobial (*a*)^39^:

$$\psi \left(a\right)=\,{\psi }_{\max }-\frac{\left({\psi }_{\max }-{\psi }_{\min }\right){\left(\frac{a}{{z{\rm{MIC}}}}\right)}^{\kappa }}{{\left(\frac{a}{{z{\rm{MIC}}}}\right)}^{\kappa }-\frac{{\psi }_{\min }}{{\psi }_{\max }}}$$

Fitting this function to the net bacterial growth rates in OriginPro 2020 (OriginLab Corporation, Northampton, MA) generates parameters *ψ*_min_ and *ψ*_max_, respectively, the minimum and maximum growth rate, *z*MIC, the pharmacodynamic minimum inhibitory concentration, and *κ*, a measure of the cooperativity. Average parameters obtained from fits of three or more independently repeated experiments were compared by one-way ANOVA with a Tukey post hoc test. Since the CFU data is log_10_ transformed, the net growth rates, are thereafter reported to three significant figures.

### *Galleria mellonella* burn wound infection model

The antimicrobial activities of WF AMPs and the clinically relevant antibiotics gentamicin, ciprofloxacin, imipenem and meropenem against a burn wound infection with *A. baumannii* ATCC 17978 were tested in *Galleria mellonella* larvae. Prior to use, *G. mellonella* were surface decontaminated by immersion in 50% ethanol for 20 s and allowed to dry. All assays were performed on three separate occasions using a group size of 10 larvae; the larvae were kept in separate petri dishes and in the dark in a static incubator at 37 °C for the duration of the experiment. Fresh agar plates of *A. baumannii* ATCC 17978 were prepared immediately prior to the experiment. Larvae were burnt using the flat head of a nail which was heated in a blue Bunsen burner flame until red hot, cooled for 15 s and superficially applied for 2 s to generate a 2 mm^2^ burn. Larvae were infected with a single colony of *A. baumannii* ATCC 17978 applied directly to the burn site. Treatment was applied topically 1 h after infection in a volume of 5 µl or sterile phosphate buffered saline as a control. Survival was monitored over 96 h.

### NMR structure determination

The NMR samples consisted of a 0.5 mM peptide solution also containing 50 mM deuterated sodium dodecyl sulphate (SDS-d_25_) with 5 mM Tris(hydroxymethyl-d_3_)-amino-d_2_-methane buffer at pH 7. 10% D_2_O containing trimethylsilyl propanoic acid (TSP) was added for the lock signal and as internal chemical shift reference. The temperature was kept constant at 298 K during the NMR experiments. NMR spectra were acquired on a Bruker Avance 600 MHz (WF1a uniquely at 700 MHz) spectrometer (Bruker, Coventry, UK) equipped with a cryoprobe. Standard Bruker TOCSY and NOESY pulse sequences were used, with water suppression using a WATERGATE 3-9-19 sequence with gradients (mlevgpph19 and noesygpph19). The ^1^H 90 degree pulse was calibrated at 37.04 kHz. The TOCSY mixing time was 80 ms, and the mixing time for the NOESY spectra was set to 200 ms. The relaxation delay was 1 s. 2048 data points were recorded in the direct dimension, and 512 data points in the indirect dimension. The spectra were processed using Bruker TOPSPIN. The free induction decay was multiplied by a shifted-sine^2^ window function. After Fourier transformation, the spectra were phase corrected, a baseline correction was applied, and spectra were calibrated to the TSP signal at 0 ppm.

Dynamo^40^ software was used for assignments and structure calculation. Inter-proton NOEs interactions were used as distance restraints in the structure calculation with the annealing protocol in Dynamo. Unambiguous NOEs only were used in this case, after being classified as strong, medium and weak on the base of their relative intensity in the NOESY spectra. Using this classification, upper limits of 0.27, 0.33 and 0.50 nm were applied, respectively, as restraint on the corresponding inter-proton distance. One thousand structures were calculated and the 100 conformers with the lowest potential energy were selected, aligned, and the root mean square deviation (RMSD) of the backbone heavy atoms calculated with respect to their average structure. Solvent molecules were not included in the calculations. Structural coordinates were deposited in the Protein Data Bank (www.rcsb.org)—see “Data availability” statement.

### Molecular dynamics simulations

Simulations were carried out on either the KCL *Rosalind* High Performance Computing (HPC) facility, or Dell Precision quad core T3400 or T3500 workstations equipped with a 1 kW Power supply (PSU) and two NVIDA PNY GeForce GTX570 or GTX580 graphics cards and occasionally the ARCHER Cray XC30 supercomputer using Gromacs 2018 or 2020^41^. The CHARMM36 all-atom force field was used in all simulations^42–44^. All membranes in this project contained a total of 512 lipids, composed either of POPE/POPG (75:25 mol:mol) or POPG. Four peptides were inserted at least 30 Angstrom above the lipid bilayer in a random position and orientation, at least 20 Angstrom apart. The starting structures were taken from the NMR calculation in SDS micelles. As previously, histidine residues were protonated^14^, but aspartate or glutamate residues were left in their anionic forms. The system was solvated with TIP3P water and Na+ ions added to neutralise. Energy minimisation was carried out at 310 K with the Nose–Hoover thermostat using the steepest descent algorithm until the maximum force was less than 1000.0 kJ/ml/nm <4000 steps). Equilibration was carried out using the NVT ensemble for 100 ps and then the NPT ensemble for 1000 ps with position restraints on the peptides. Hydrogen-containing bond angles were constrained with the LINCS algorithm. Final simulations were run in the NPT ensemble using 2 fs intervals, with trajectories recorded every 2 ps. All simulations were run in triplicate for a total of 200 ns with peptides inserted at different positions and orientations, giving a total of approximately 10.8 µs simulation. Torsion angles are circular quantities, and the circular mean of psi or phi angles may be calculated as follows:

$$\bar{\psi }=\,{atan}2\,\left(\frac{1}{n}\mathop{\sum }\limits_{j=1}^{n}{{\sin }}{\psi }_{j},\frac{1}{n}\,\mathop{\sum }\limits_{j=1}^{n}{{\cos }}{\psi }_{j}\right)$$

Similarly, the associated circular variance for psi or phi angles is calculated as follows:

Var (*ψ*) = 1 − *R*_av_

with *R* being given by:

$${R}^{2}=\,{\left(\mathop{\sum }\limits_{i=1}^{n}{{{\cos }}\psi }_{i}\right)}^{2}+\,{\left(\mathop{\sum }\limits_{i=1}^{n}{{{\sin }}\psi }_{i}\right)}^{2}$$

### Liposome preparation and circular dichroism spectroscopy

Far-UV CD spectra of the peptides bound to small unilamellar vesicles (SUV) were obtained using a Chirascan Plus spectrometer (Applied Photophysics, Leatherhead, UK) with samples maintained at 310 K. To prepare SUV, lipid powders were solubilized in chloroform and dried under rotor-evaporation. To completely remove the organic solvent, the lipid films were left overnight under vacuum and hydrate in 5 mM Tris buffer (pH 7.0). The lipid suspension was subjected to 5 rapid freeze-thaw cycles for further sample homogenisation. POPE/POPG (75:25, mol:mol) and POPG SUVs were obtained by sonicating the lipid suspension on Soniprep 150 (Measuring and Scientific Equipment, London, UK) for 3 × 5 min with amplitude of 6 microns in the presence of ice to avoid lipid degradation. The SUVs were stored at 4 °C and used within 5 days of preparation. Far-UV CD spectra were recorded from 260 to 190 nm. SUV suspension was added to a 0.5 mm cuvette at a final concentration of 3.0 mM and then a few μl of a concentrated peptide solution were added and thoroughly mixed to give a final peptide concentration of 15 μM. The same experimental conditions were used to investigate peptide secondary structure in SDS micelles, while the SDS micelles concentration was 5 mM with 50 µM peptide. In processing, a spectrum of the peptide free lipid suspension or SDS solution was subtracted and Savitsky–Golay smoothing with a convolution width of 5 points applied.

### Electrophysiology experiments (patch-clamp)

As in our earlier work^18^, lipids with diphytanoyl chains are used here to form giant unilamellar vesicles (GUV). GUVs composed of DPhPE/DPhPG (60:40, mol:mol) and DPhPG were prepared in the presence of 1 M sorbitol by the electroformation method in an indium-tin oxide (ITO) coated glass chamber connected to the Nanion Vesicle Prep Pro setup (Nanion Technologies GmbH, Munich, Germany) using a peak-to-peak AC voltage of 3 V, at a frequency of 5 Hz, for 120 and 140 min, respectively, at 37 °C. Bilayers were formed by adding the GUV solution to a buffer containing 250 mM KCl, 50 mM MgCl_2_ and 10 mM Hepes (pH 7.00) onto an aperture in a borosilicate chip (Port-a-Patch®; Nanion Technologies) and applying 70–90 mbar negative pressure resulting in a solvent-free membrane with a resistance in the GΩ range. After formation, a small amount of peptide stock solution (in water) was added to 50 μl of buffer solution to obtain its active concentration. All the experiments were carried on with a positive holding potential of 50 mV. The active concentration, the concentration at which the peptide first showed membrane activity, for each peptide was obtained through a titration performed in the same conditions. For all the experiments a minimum of 6 concordant repeats was done. Current traces were recorded at a sampling rate of 50 kHz using an EPC-10 amplifier from HEKA Elektronik (Lambrecht, Germany). The system was computer controlled by the PatchControl™ software (Nanion) and GePulse (Michael Pusch, Genoa, Italy). The data were filtered using the built-in Bessel filter of the EPC-10 at a cut-off frequency of 10 kHz. The experiments were performed at room temperature. Data analysis was performed with the pClamp 10 software package (Axon Instruments).

### Reporting summary

Further information on research design is available in the Nature Research Reporting Summary linked to this article.

## Supplementary information

Supplementary Material

REPORTING SUMMARY

## Data Availability

Supplementary Information includes more extensive analysis of the MD simulation data, circular dichroism experiments and further analysis of the patch-clamp data. Structural coordinates were deposited in the Protein Data Bank (www.rcsb.org) and Biological Magnetic Resonance Bank (BMRB; www.bmrb.wisc.edu) under accession codes of 6S2D, 6RYQ, 6RY9, 6RZ1 and 6RZC (PDB) and 34416, 34411, 34410, 34412, and 34413 (BMRB) for WF1, WF1a, WF1a-1, WF3 and WF4, respectively. In addition to the structural coordinates, the data sets generated during and/or analysed during the current study are available from the corresponding author on reasonable request.

## References

[1] Yu, G., Baeder, D. Y., Regoes, R. R. & Rolff, J. Combination effects of antimicrobial peptides. *Antimicrob. Agents Chemother.* **60**, 1717–1724 (2016). 26729502 10.1128/AAC.02434-15 PMC4775937

[2] Yu, G., Baeder, D. Y., Regoes, R. R. & Rolff, J. Predicting drug resistance evolution: insights from antimicrobial peptides and antibiotics. *Proc. Biol. Sci.* **285**, 20172687 (2018). 29540517 10.1098/rspb.2017.2687 PMC5879628

[3] Kubicek-Sutherland, J. Z. et al. Antimicrobial peptide exposure selects for *Staphylococcus aureus* resistance to human defence peptides. *J. Antimicrob. Chemother.* **72**, 115–117 (2017). 27650186 10.1093/jac/dkw381 PMC5161045

[4] Jangir, P. K., Ogunlana, L. & MacLean, R. C. Evolutionary constraints on the acquisition of antimicrobial peptide resistance in bacterial pathogens. *Trends Microbiol.* **29**, 1058–1061 (2021). 33836929 10.1016/j.tim.2021.03.007

[5] Spohn, R. et al. Integrated evolutionary analysis reveals antimicrobial peptides with limited resistance. *Nat. Commun.* **10**, 4538 (2019). 31586049 10.1038/s41467-019-12364-6 PMC6778101

[6] Thomas, J. K. et al. Pharmacodynamic evaluation of factors associated with the development of bacterial resistance in acutely ill patients during therapy. *Antimicrob. Agents Chemother.* **42**, 521–527 (1998). 9517926 10.1128/aac.42.3.521 PMC105492

[7] Ferguson, P. N. et al. Temporin B forms hetero-oligomers with temporin L, modifies its membrane activity, and increases the cooperativity of its antibacterial pharmacodynamic profile. *Biochemistry* **61**, 1029–1040 (2022). 35609188 10.1021/acs.biochem.1c00762 PMC9178791

[8] Cole, A. M., Weis, P. & Diamond, G. Isolation and characterization of pleurocidin, an antimicrobial peptide in the skin secretions of Winter Flounder. *J. Biol. Chem.* **272**, 12008–12013 (1997). 9115266 10.1074/jbc.272.18.12008

[9] Saint, N., Cadiou, H., Bessin, Y. & Molle, G. Antibacterial peptide pleurocidin forms ion channels in planar lipid bilayers. *Biochim. Biophys. Acta* **1564**, 359–364 (2002). 12175918 10.1016/s0005-2736(02)00470-4

[10] Yoshida, K. et al. Interaction of pleurocidin and its analogs with phospholipid membrane and their antibacterial activity. *J. Peptide Res.* **57**, 119–126 (2001). 11168895 10.1034/j.1399-3011.2001.00802.x

[11] Kozlowska, J. et al. Combined systems approaches reveal highly plastic responses to antimicrobial peptide challenge in *Escherichia coli*. *PLoS Pathogens* **10**, e1004104 (2014). 24789011 10.1371/journal.ppat.1004104 PMC4006907

[12] Patrzykat, A., Friedrich, C. L., Zhang, L., Mendoza, V. & Hancock, R. E. W. Sublethal concentrations of Pleurocidin-derived antimicrobial peptides inhibit macromolecular synthesis in *Escherichia coli*. *Antimicrob. Agents Chemother.* **46**, 605–614 (2002). 11850238 10.1128/AAC.46.03.605-614.2002 PMC127508

[13] Lan, Y. et al. Structural contributions to the intracellular targeting strategy of antimicrobial peptides. *Biochim. Biophys. Acta* **1798**, 1934–1943 (2010). 20637722 10.1016/j.bbamem.2010.07.003 PMC3309561

[14] Manzo, G. et al. A Pleurocidin analogue with greater conformational flexibility, enhanced antimicrobial potency and in vivo therapeutic efficacy. *Commun. Biol.* **3**, 697 (2020). 33247193 10.1038/s42003-020-01420-3 PMC7699649

[15] Czaplewski, L. et al. Alternatives to antibiotics—a pipeline portfolio review. *Lancet. Infect. Dis.* **16**, 239–251 (2016). 26795692 10.1016/S1473-3099(15)00466-1

[16] Douglas, S. E., Gallant, J. W., Gong, Z. & Hew, C. Cloning and developmental expression of a family of pleurocidin like antimicrobial peptides from winter founder, *Pleuronectes americanus* (Walbaum). *Dev. Compar. Immunol.* **25**, 137–147 (2001). 10.1016/s0145-305x(00)00052-5 11113283

[17] Patrzykat, A., Gallant, J. W., Seo, J.-L., Pytyck, J. & Douglas, S. E. Novel antimicrobial peptides derived from flatfish genes. *Antimicrob. Agents Chemother.* **47**, 2464–2470 (2003). 12878506 10.1128/AAC.47.8.2464-2470.2003 PMC166104

[18] Fratini, F. et al. A novel interpretation of the Fractional Inhibitory Concentration Index: the case *Origanum vulgare* L. and *Leptospermum scoparium* J. R. et G. Forst essential oils against *Staphylococcus aureus* strains. *Microbiol. Res.* **195**, 11–17 (2017). 28024521 10.1016/j.micres.2016.11.005

[19] Tekin, E. et al. Prevalence and patterns of higher-order drug interactions in *Escherichia coli*. *npj Syst. Biol. Appl.* **4**, 31 (2018). 30181902 10.1038/s41540-018-0069-9 PMC6119685

[20] Manzo, G. et al. Temporin L and aurein 2.5 have identical conformations but subtly distinct membrane and antibacterial activities. *Sci. Rep.* **9**, 10934 (2019). 31358802 10.1038/s41598-019-47327-w PMC6662694

[21] Manzo, G. et al. Minor sequence modifications in temporin B cause drastic changes in antibacterial potency and selectivity by fundamentally altering membrane activity. *Sci. Rep.* **9**, 1385 (2019). 30718667 10.1038/s41598-018-37630-3 PMC6362004

[22] Di Blasio, S. et al. Bolaamphiphile analogues of 12-bis-THA Cl2 are potent antimicrobial therapeutics with distinct mechanisms of action against bacterial, mycobacterial, and fungal pathogens. *mSphere* **8**, e00508–e00522 (2022). 36511707 10.1128/msphere.00508-22 PMC9942557

[23] Maslova, E. et al. An invertebrate burn wound model that recapitulates the hallmarks of burn trauma and infection seen in mammalian models. *Front. Microbiol.* **11**, 998 (2020). 32582051 10.3389/fmicb.2020.00998 PMC7283582

[24] Sani, M.-A. & Separovic, F. How membrane-active peptides get into lipid membranes. *Acc. Chem. Res.* **49**, 1130–1138 (2016). 27187572 10.1021/acs.accounts.6b00074

[25] Strandberg, E. et al. Influence of hydrophobic residues on the activity of the antimicrobial peptide Magainin 2 and its synergy with PGLa. *J. Pept. Sci.* **21**, 436–445 (2015). 25898805 10.1002/psc.2780

[26] Zerweck, J. et al. Homo- and heteromeric interaction strengths of the synergistic antimicrobial peptides PGLa and Magainin 2 in membranes. *Eur. Biophys. J.* **45**, 535–547 (2016). 27052218 10.1007/s00249-016-1120-7

[27] Zerweck, J. et al. Molecular mechanism of synergy between the antimicrobial peptides PGLa and Magainin 2. *Sci. Rep.* **7**, 13153 (2017). 29030606 10.1038/s41598-017-12599-7 PMC5640672

[28] Aisenbrey, C., Amaro, M., Pospíšil, P., Hof, M. & Bechinger, B. Highly synergistic antimicrobial activity of Magainin 2 and PGLa peptides is rooted in the formation of supramolecular complexes with lipids. *Sci. Rep.* **10**, 11652 (2020). 32669585 10.1038/s41598-020-68416-1 PMC7363891

[29] Ma, W. et al. Individual roles of peptides PGLa and Magainin 2 in synergistic membrane poration. *Langmuir* **36**, 7190–7199 (2020). 32529830 10.1021/acs.langmuir.0c00194

[30] Amos, S.-B. T. A. et al. Antimicrobial peptide potency is facilitated by greater conformational flexibility when binding to Gram-negative bacterial inner membranes. *Sci. Rep.* **6**, 37639 (2016). 27874065 10.1038/srep37639 PMC5118786

[31] Lopes, J. L. S., Miles, A. J., Whitmore, L. & Wallace, B. A. Distinct circular dichroism spectroscopic signatures of polyproline II and unordered secondary structures: applications in secondary structure analyses. *Protein Sci.* **23**, 1765–1772 (2014). 25262612 10.1002/pro.2558 PMC4253816

[32] Mason, A. J., Marquette, A. & Bechinger, B. Zwitterionic phospholipids and sterols modulate antimicrobial peptide-induced membrane destabilization. *Biophys. J.* **93**, 4289–4299 (2007). 17766347 10.1529/biophysj.107.116681 PMC2098721

[33] Douglas, S. E., Gallant, J. W., Gong, Z. & Hew, C. Cloning and developmental expression of a family of pleurocidin-like antimicrobial peptides from winter flounder, *Pleuronectes americanus* (Walbaum). *Dev. Comp. Immunol.* **25**, 137–147 (2001). 11113283 10.1016/s0145-305x(00)00052-5

[34] Lazzaroa, B. P., Zasloff, M. & Rolff, J. Antimicrobial peptides: application informed by evolution. *Science* **368**, 487 (2020). 10.1126/science.aau5480 PMC8097767 32355003

[35] Tangy, F., Moukkadem, M., Vindimian, E., Capmau, M. L. & Le Goffic, F. Mechanism of action of gentamicin components. Characteristics of their binding to *Escherichia coli* ribosomes. *Eur. J. Biochem.* **147**, 381–386 (1985). 3882427 10.1111/j.1432-1033.1985.tb08761.x

[36] El Shazeley, B., Yu, G., Johnston, P. R. & Rolff, J. Resistance evolution against antimicrobial peptides in *Staphylococcus aureus* alters pharmacodynamics beyond the MIC. *Front. Microbiol.* **11**, 103 (2020). 32117132 10.3389/fmicb.2020.00103 PMC7033599

[37] Wiegand, I., Hilpert, K. & Hancock, R. E. Agar and broth dilution methods to determine the minimal inhibitory concentration (MIC) of antimicrobial substances. *Nat. Protoc.* **3**, 163–175 (2008). 18274517 10.1038/nprot.2007.521

[38] Herigstad, B., Hamilton, M. & Heersink, J. How to optimize the drop plate method for enumerating bacteria. *J. Microb. Methods.* **44**, 121–129 (2001). 10.1016/s0167-7012(00)00241-4 11165341

[39] Regoes, R. R. et al. Pharmacodynamic functions: a multiparameter approach to the design of antibiotic treatment regimens. *Antimicrob. Agents Chemother.* **48**, 3670–3676 (2004). 15388418 10.1128/AAC.48.10.3670-3676.2004 PMC521919

[40] NIDDK, NIH. Dynamo software: the NMR molecular dynamics and analysis system. http://spin.niddk.nih.gov/NMRPipe/dynamo (2023).

[41] Abraham, M. J. et al. GROMACS: high performance molecular simulations through multi-level parallelism from laptops to supercomputers. *SoftwareX* **1**, 19–25 (2015).

[42] Best, R. B. et al. Optimization of the additive CHARMM all-atom protein force field targeting improved sampling of the backbone φ, ψ and side-chain χ1 and χ2 dihedral angles. *J. Chem. Theory Comput.* **8**, 3257–3273 (2012). 23341755 10.1021/ct300400x PMC3549273

[43] Huang, J. & MacKerell, A. D. CHARMM36 all-atom additive protein force field: validation based on comparison to NMR data. *J. Comput. Chem.* **34**, 2135–2145 (2013). 23832629 10.1002/jcc.23354 PMC3800559

[44] Lee, J. et al. CHARMM-GUI input generator for NAMD, GROMACS, AMBER, OpenMM, and CHARMM/OpenMM simulations using the CHARMM36 additive force field. *J. Chem. Theory Comput.* **12**, 405–413 (2016). 26631602 10.1021/acs.jctc.5b00935 PMC4712441

[45] White, S. H. & Wimley, W. C. Hydrophobic interactions of peptides with membrane interfaces. *Biochim. Biophys. Acta* **1376**, 339–352 (1998). 9804985 10.1016/s0304-4157(98)00021-5

