# Supplementary material for: Synergy between Winter Flounder antimicrobial peptides
Source: NPJ Antimicrob Resist. 2023 Aug 10;1:8. doi: 10.1038/s44259-023-00010-7 (PMC11057203; doi:10.1038/s44259-023-00010-7)
Supplement: Supplementary file 1 — Supplementary Material [file 44259_2023_10_MOESM1_ESM.pdf]

Synergy between Winter Flounder Antimicrobial Peptides

Maria Clarke[1], Charlotte K. Hind[2], Philip M. Ferguson[1], Giorgia Manzo[1], Bhumil Mistry[1], Bingkun Yue[1], Janis Romanopulos[1], Melanie Clifford[2], Tam T. Bui[3], Alex F. Drake[3], Christian D. Lorenz[4]*, J. Mark Sutton[1,2]* and A. James Mason[1]*

[1]Institute of Pharmaceutical Science, School of Cancer & Pharmaceutical Science, King's College London, Franklin-Wilkins Building, 150 Stamford Street, London, SE1 9NH, United Kingdom

[2]Technology Development Group, UK Health Security Agency, Research and Evaluation, Porton Down, Salisbury, SP4 0JG, United Kingdom

[3]Centre for Biomolecular Spectroscopy and Randall Division of Cell and Molecular Biophysics, King's College London, New Hunt's House, London SE1 1UL, United Kingdom

[4]Department of Physics, King's College London, London WC2R 2LS, United Kingdom

Supplementary Material

Isolate	Pleurocidin	D-pleurocidin	D-WF1a-1
	Gram-negative		
Klebsiella pneumoniae NCTC 13368	4	2	2
Klebsiella pneumoniae M6	1	2	2
Acinetobacter baumannii AYE	2	2	2
Acinetobacter baumannii ATCC 17978	1	1	1
Pseudomonas aeruginosa PAO1	4	2	2
Pseudomonas aeruginosa NCTC 13437	16	8	8
Escherichia coli NCTC 12923	1	1	2
	Gram-positive		
Staphylococcus aureus 1199B	4	16	8
Staphylococcus aureus USA300	4	32	8
EMR *Staphylococcus aureus*-15 NCTC 13616	4	16	4
Staphylococcus aureus NCTC 6571	4	16	8
VS *Enterococcus faecalis* NCTC 775	32	16	64
VR *Enterococcus faecalis* NCTC 12201	32	64	64
VR *Enterococcus faecium* NCTC 12204	32	16	64
	Extended *Pseudomonas aeruginosa*		
P. aeruginosa CAS2	16	8	**2**
P. aeruginosa CAS3	16	**4**	**4**
P. aeruginosa CAS4	>128	**8**	**4**
P. aeruginosa GH12	32	**4**	**4**
P. aeruginosa RP73	16	16	8
P. aeruginosa 372261	16	**4**	**4**

Supplementary Table 1. Antimicrobial activity of D-enantiomers. EMR – epidemic methicillin resistant; VS – vancomycin sensitive; VR – vancomycin resistant. Values are given for peptides tested in Mueller-Hinton broth. Grey or bold values indicate respectively a significant (factor of >2) reduction or improvement in potency relative to pleurocidin.

Four-way combinations				
Condition	% Inhibition	Additive threshold	Bliss Net	p value
**WF1/WF1a/WF1a1/WF2**	**99.53**	**33.21**	**66.32**	**0.0003**
**WF1/WF1a/WF1a1/WF3**	**100.00**	**26.53**	**73.51**	**<0.0001**
**WF1/WF1a/WF1a1/WF4**	61.90	27.69	34.20	0.2011
**WF1/WF1a/WF2/WF3**	**99.94**	**16.69**	**83.25**	**<0.0001**
**WF1/WF1a/WF2/WF4**	**88.78**	**17.85**	**70.93**	**0.0001**
**WF1/WF1a/WF3/WF4**	34.62	11.18	23.44	0.7432
**WF1/WF1a1/WF2/WF3**	**99.80**	**28.66**	**71.14**	**0.0001**
**WF1/WF1a1/WF2/WF4**	60.11	29.82	30.29	0.3597
**WF1/WF1a1/WF3/WF4**	**79.12**	**23.14**	**55.98**	**0.0030**
**WF1/WF2/WF3/WF4**	**99.61**	**13.30**	**86.30**	**<0.0001**
**WF1a/WF1a1/WF2/WF3**	**99.50**	**27.74**	**71.76**	**0.0001**
**WF1a/WF1a1/WF2/WF4**	**98.82**	**28.90**	**69.91**	**0.0002**
**WF1a/Wf1a1/WF3/WF4**	**87.05**	**22.22**	**64.82**	**0.0005**
**WF1a/WF2/WF3/WF4**	**94.84**	**12.39**	**82.45**	**<0.0001**
**WF1a1/WF2/WF3/WF4**	**99.34**	**24.35**	**74.99**	**<0.0001**

Supplementary Table 2. Bliss independence model of four-way combinations. The percentage inhibition of *K. pneumoniae* NCTC 13368 in MHB grown in the presence of four-way combinations of the six WF AMPs according to the method of Tekin et al. Subtraction of the additive threshold – inhibition expected from adding the contributions from non-interacting WF AMPs – from the inhibition achieved provides the Bliss net inhibition. Two-Way ANOVA with Šídák's multiple comparisons test identifies conditions (shown in bold) where synergy is significant relative to the purely additive combination of each individual WF AMP but does not account for lower order synergy (and hence is not necessarily emergent).

Five-way combinations				
Condition	% Inhibition	Additive threshold	Bliss Net	p value
**WF1/WF1a/WF1a1/WF2/WF3**	**98.25**	**33,21**	**65.04**	**0.0171**
**WF1a/WF1a1/WF2/WF3/WF4**	**99.07**	**28.90**	**70.17**	**0.0097**
**WF1a1/WF2/WF3/WF4/WF1**	**100.10**	**29.82**	**70.31**	**0.0095**
**WF2/WF3/WF4/WF1/WF1a**	**99.41**	**17.85**	**81.55**	**0.0028**
**WF3/WF4/WF1/WF1a/WF1a1**	**99.59**	**27.69**	**71.90**	**0.0080**
**WF4/WF1/WF1a/WF1a1/WF2**	**97.90**	**34.37**	**63.54**	**0.0202**
**Six-way combination**				
**WF1/WF1a/WF1a1/WF2/WF3/WF4**	**100.00**	**34.37**	**65.64**	**0.0160**

Supplementary Table 3. Bliss independence model of five-way and six-way combinations. The percentage inhibition of *K. pneumoniae* NCTC 13368 in MHB grown in the presence of six-way or five-way combinations of the six WF AMPs according to the method of Tekin et al. Subtraction of the additive threshold – inhibition expected from adding the contributions from non-interacting WF AMPs – from the inhibition achieved provides the Bliss net inhibition. Two-Way ANOVA with Šídák's multiple comparisons test identifies conditions (shown in bold) where synergy is significant relative to the purely additive combination of each individual WF AMP but does not account for lower order synergy (and hence is not necessarily emergent).

Media	Isolate / peptide	Pleurocidin MIC	Pleurocidin MIC (combination)	D-WF1a MIC	D-WF1a MIC (Combination)	FIC_{min} (Mean ± SE)
MHB	EMRSA-15 / D-pleurocidin	8-16	4-6	512	256	0.917 ± 0.125
	EMRSA-15 / D-pleurocidin-KR	2	0.5-0.94	512	32-256	0.678 ± 0.109
	P. aeruginosa RP73 / D- pleurocidin	8-16	3-3.5	512	64-128	**0.391 ± 0.031**
	P. aeruginosa RP73 / D-pleurocidin-KR	8-16	3-3.5	512	64-128	0.542 ± 0.094
RPMI	EMRSA-15 / D-pleurocidin	0.5-1	0.06, 0.12, 0.12	64	4, 8, 16	**0.290 ± 0.092**
	EMRSA-15 / D-pleurocidin-KR	0.5	0.06-0.12	64	4-8	**0.290 ± 0.030**
	P. aeruginosa RP73 / D- pleurocidin	16-32	1:75-3.5	512	64	**0.234 ± 0.000**
	P. aeruginosa RP73 / D-pleurocidin-KR	32	6-7	512	64-128	**0.375 ± 0.031**

Supplementary Table 4. Synergy between D-enantiomers. Synergy between D-WF1a and either D-pleurocidin or D-pleurocidin-KR was determined using checkerboard assays. Concordant MICs are presented (µg/ml) and FICmin are the average of three independent repeats. Conditions where a ≥ 4-fold reduction in both components' MIC is consistently observed are indicated in bold

Panel	Drug	Top concentration (µg/mL)	
		100x	200x
(A) AB17978 in MHB	Meropenem	6	-
	Ciprofloxacin	50	-
	Imipenem	50	-
	Gentamicin	50	-
	WF2	200	-
	WF1a/WF2	400/25	-
	WF3/WF2	200/25	-
	WF4	300	-
	WF3/WF4	200/50	-
(C) AYE in MHB	Imipenem	-	200
	WF2	200	-
	WF1a/WF2	400/25	-
	WF3/WF2	200/25	-
	WF4	400	-
	WF3/WF4	200/50	-
(E) EMRSA15 in RPMI	D-WF2	100	-
	WF2	800	-
	D-WF1a/D-WF2	1600/12.5	-
	Tobramycin	-	200
	Gentamicin	-	50
	Daptomycin	-	100

Supplementary Table 5. Antibiotic top-concentrations used in *in vitro* PD experiments. This table shows the top concentrations, either 100 or 200 times the respective MICs given in Tables 2 (AMPs in isolation) or Table 4 (AMPs in combination), that are used in the *in vitro* PD experiments described in Figure 1. The x-axis in Panels A, B and C therefore correspond to fractions or multiples of the MIC up to these top concentrations.

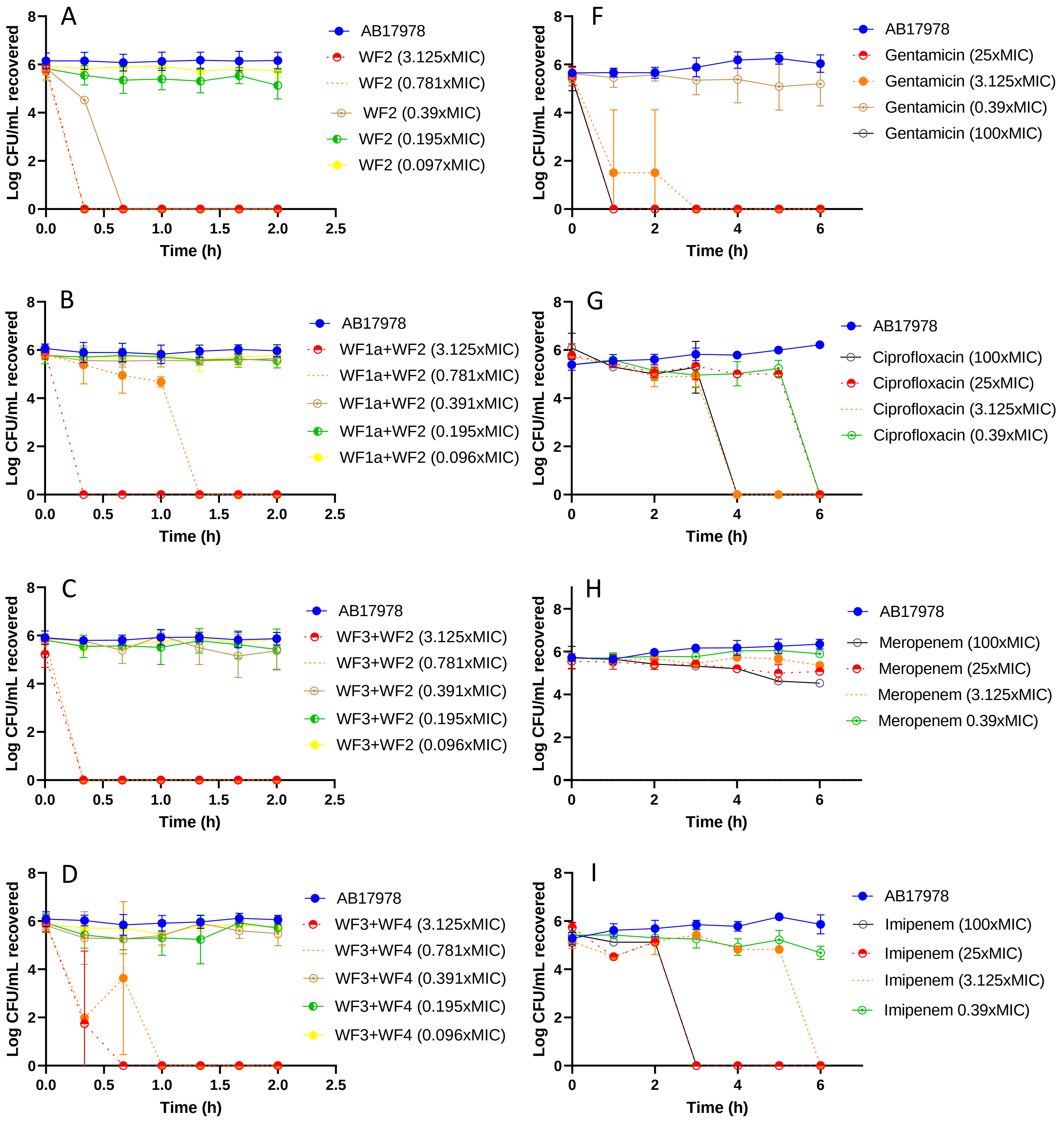

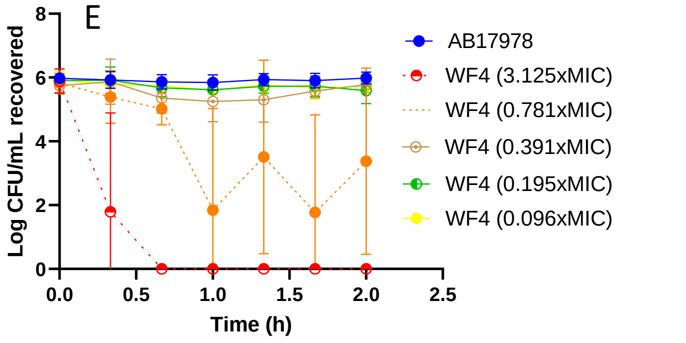

Supplementary Figure 1. Example time-kill curves reconstructed from *in vitro* PD studies. Colony forming units per ml of *A. baumannii* 17978 are shown as a function of time for AMPs and combinations sampled every 20 minutes for 2 hours (A-E), and for antibiotics sampled every hour for six hours (F-I). PD curves are constructed from three independent time-kill experiments conducted at ten different concentrations. AMPs are rapidly bactericidal while meropenem, in contrast with imipenem, is bacteriostatic In these conditions

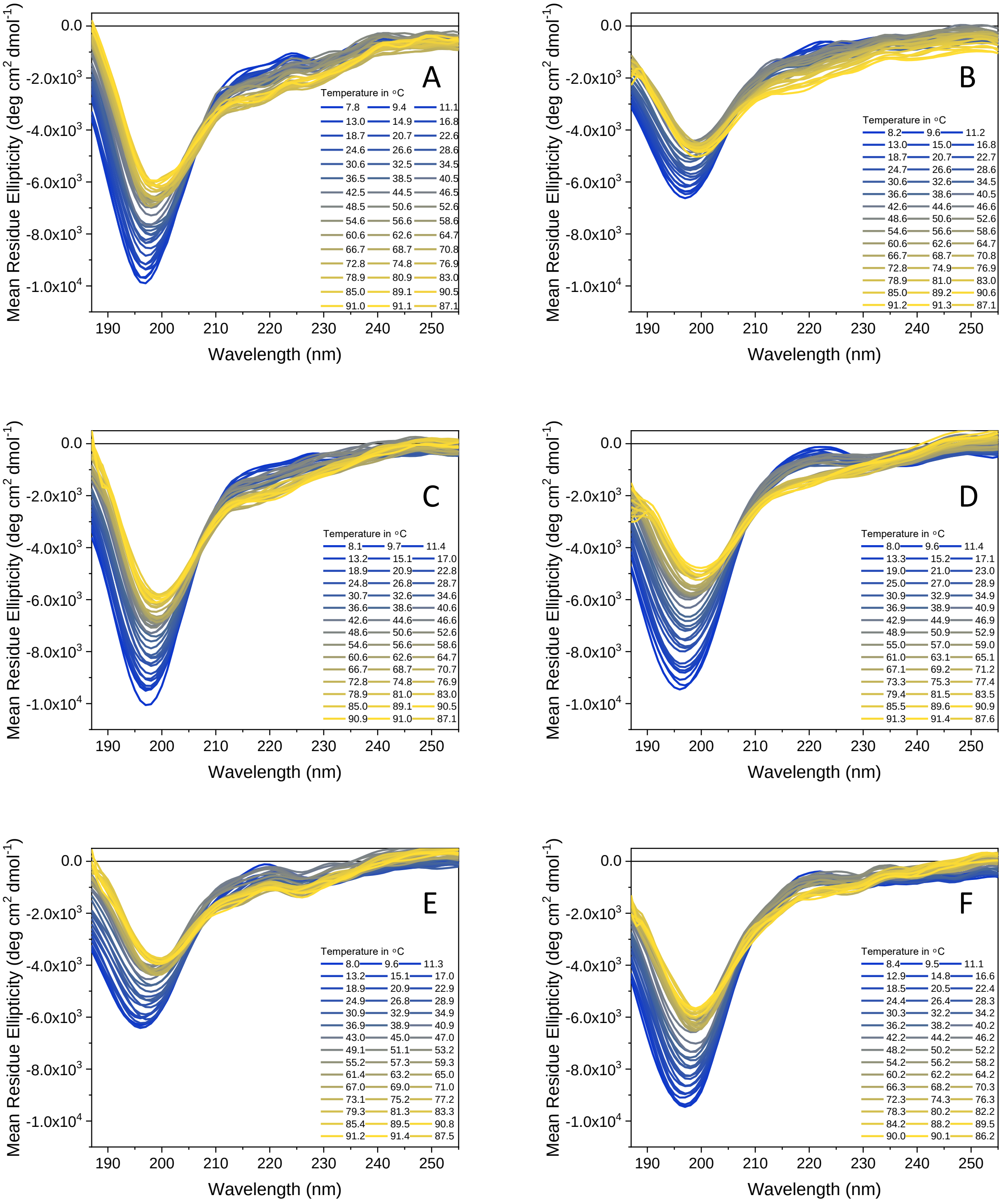

Supplementary Figure 2. WF peptides adopt ordered conformations in aqueous solution that melt at elevated temperatures. Far-UV circular dichroism spectra of Winter Flounder peptides were obtained in duplicate in 5 mM Tris pH 7.0 buffer from 4 - 91˚C in 2°C increments. A: WF1, B: WF1a, C: WF1a1, D: WF2, E:WF3, F:WF4

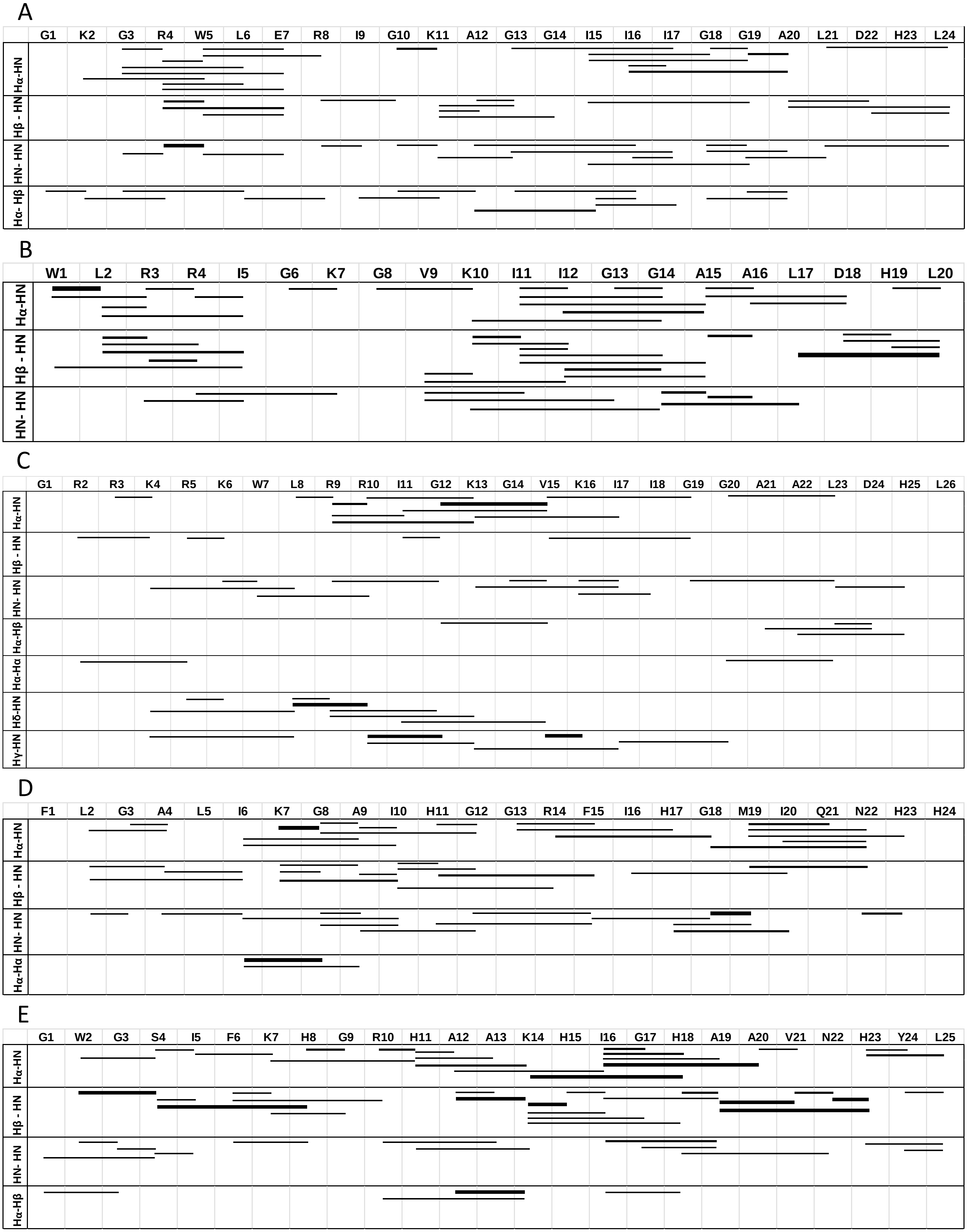

Supplementary Figure 3. Summary of ^{1}H-^{1}H NOEs from structure determination of WF AMPs. Short and medium range interproton NOEs found for WF1 (A), WF1a (B), WF1a-1 (C), WF3 (D) and WF4 (E) in SDS-d_{25} micelles. NOEs are reported as lines connecting the two involved residues and the thickness of the line is proportional to the relative intensity (strong, medium, weak) of the corresponding NOESY cross-peak.

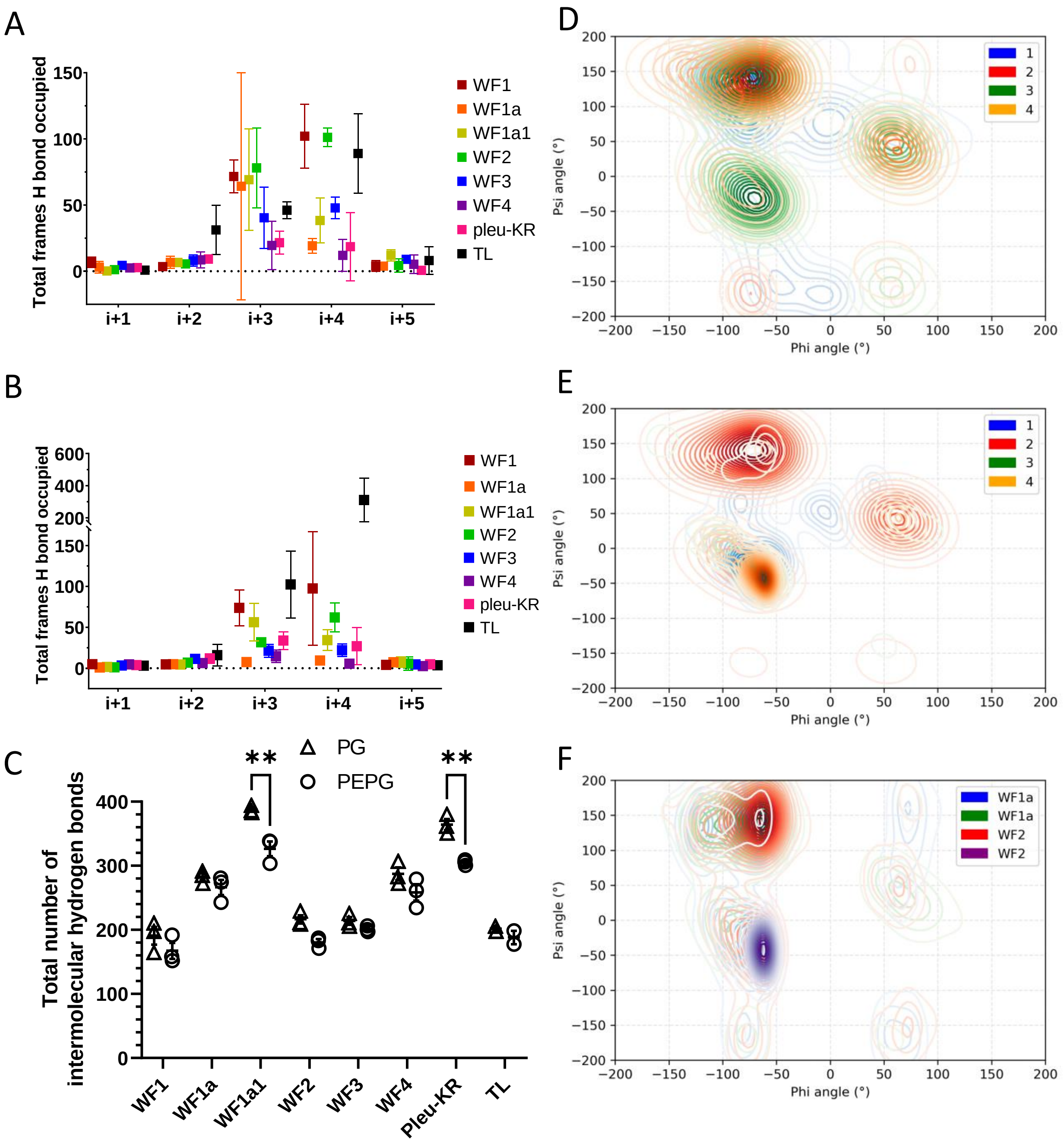

Supplementary Figure 4. MD simulations indicate WF peptides adopt both α-helix and P_{II} conformations. Total AMP intra-molecular (A, B) and AMP to lipid inter-molecular (C) H-bonding is plotted for six WF peptides, pleurocidin-KR and temporin L inserting into POPG (A), POPE/POPG (B) or both (C) bilayers. For intramolecular hydrogen bonding the number of frames where the hydrogen bond is plotted as the average and SEM of three independent replicates, normalised to the number of residues in each peptide. For peptide-lipid intermolecular hydrogen bonding there is no normalisation. Ramachandran plots (D-F) show the average psi and phi angles for four individual WF1a peptides (D), four individual WF2 peptides (E) or each of two WF1a or WF2 peptides (F) inserting in POPE/POPG bilayers (B).

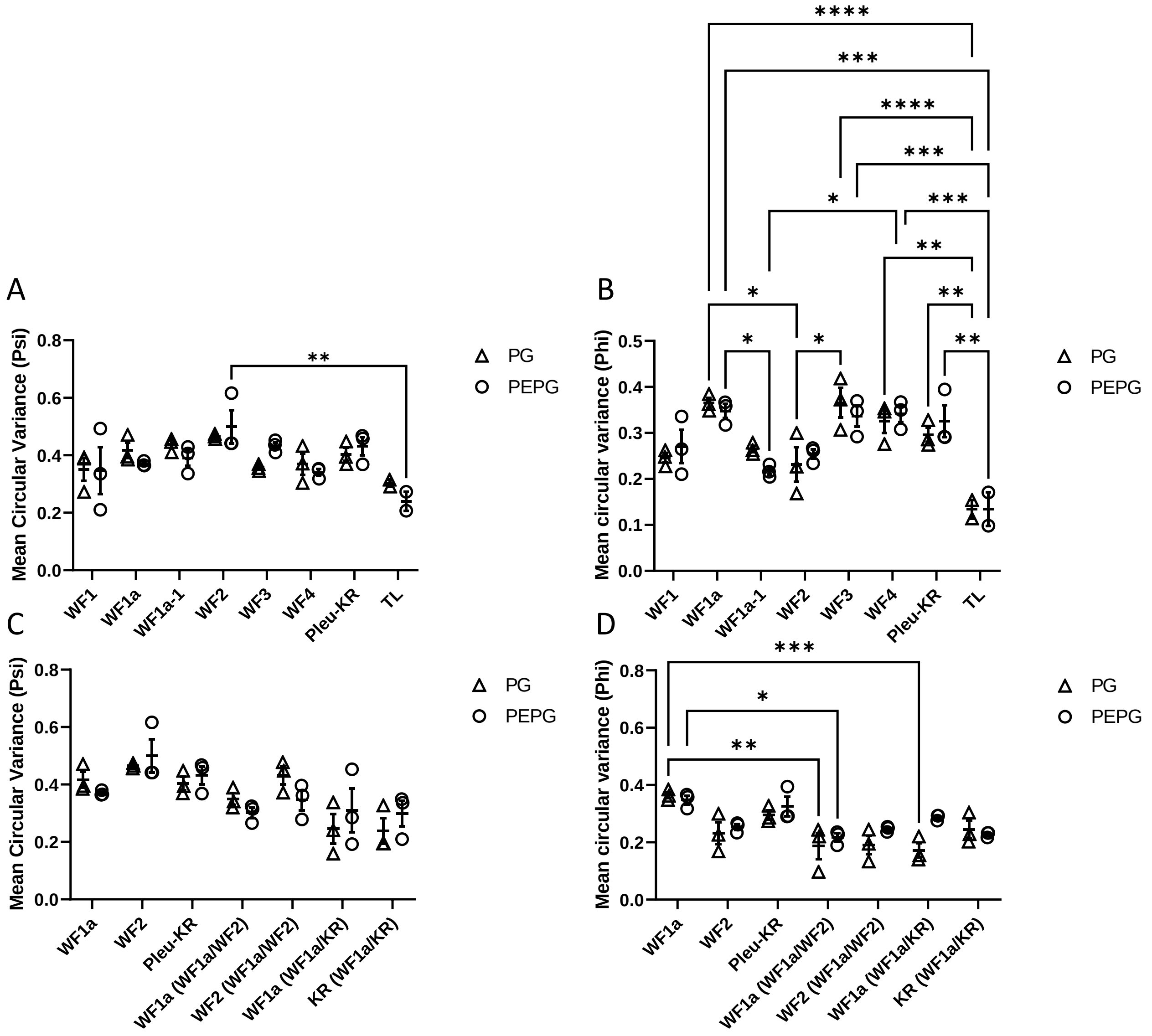

Supplementary Figure 5. Conformational flexibility in MD simulations. Circular variance over the last 100 ns of the 200 ns simulations is shown for simulations of four peptides (A, B) or, for the combinations 2 + 2 peptides (C, D), inserting in either POPG or POPE/POPG bilayers with each point representing the average of the four peptides (n = 3 except temporin L where n = 2) with error bars the SEM.

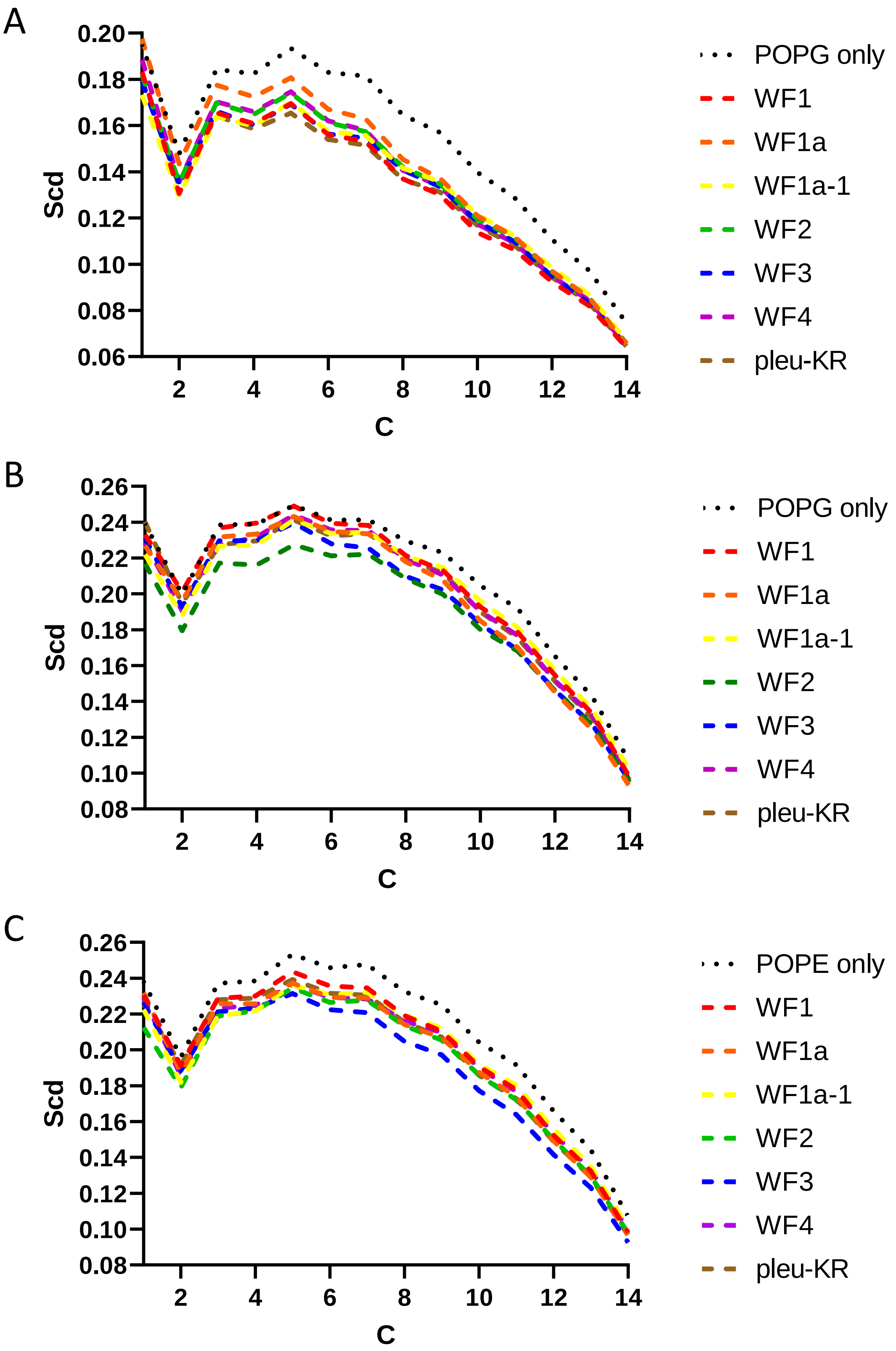

Supplementary Figure 6. MD simulation – WF peptides disorder anionic bilayers upon penetration. Lipid acyl chain order parameters for lipids within 4 Å of a peptide shown as averages of the three replicates for POPG bilayers (A) and either POPG (B) or POPE (C) in mixed POPE/POPG bilayers.

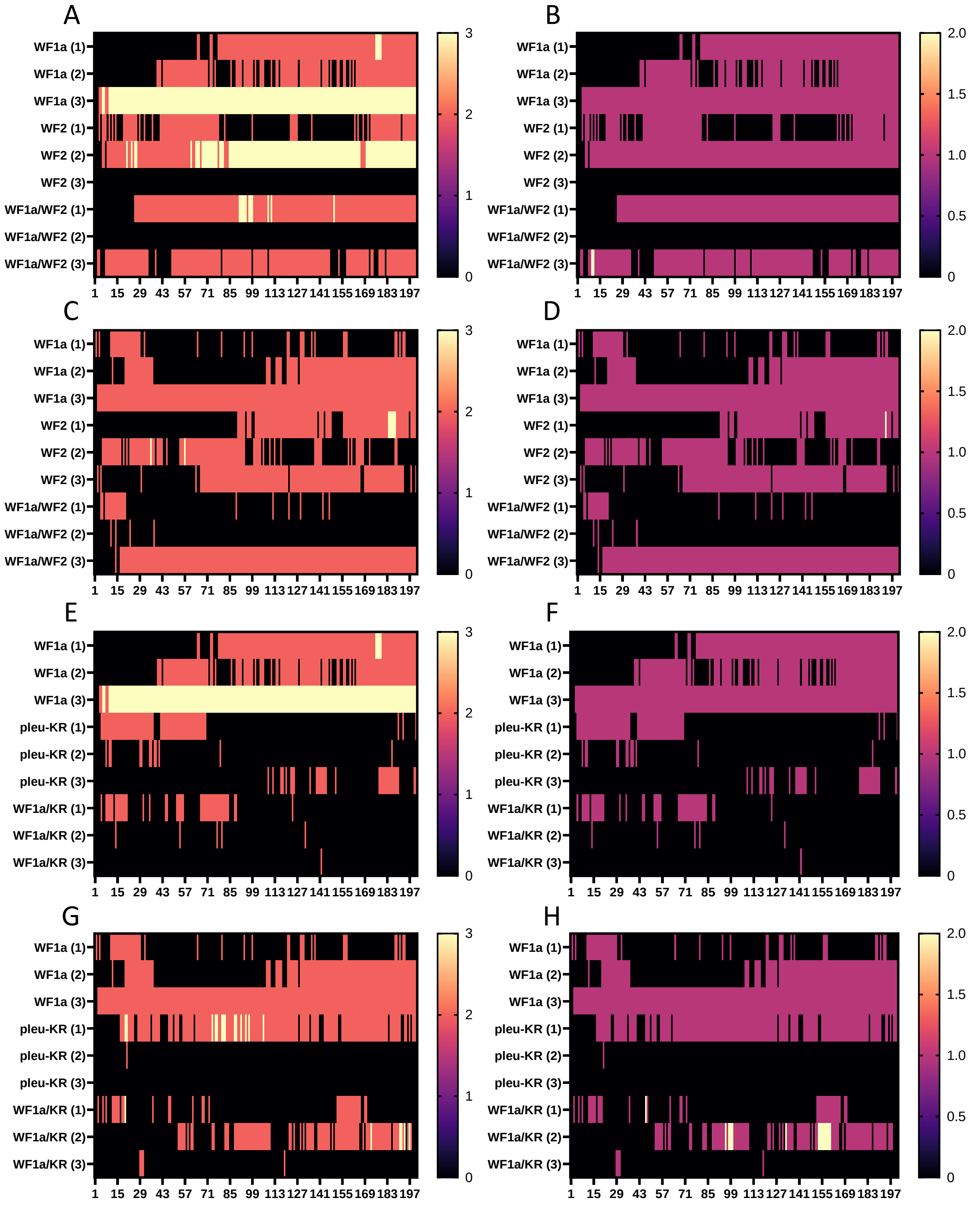

Supplementary Figure 7. Aggregation analyses for WF AMPs in the MD simulations. The number of peptides that cluster together at any one time (A, C, E, G) and the number of such clusters at any one time (B, D, F, H) are shown as a function of time over the duration of the 200 ns simulation for the indicated peptides when binding and inserting into POPG (A,B, E, F) or POPE/POPG (C, D, G, F) bilayers. There are four WF AMPs in each simulation – either four WF1a, WF2 or pleurocidin-KR or, for the combinations two WF1a plus two WF2 or two WF1a plus two pleurocidin-KR. Dimers are formed frequently but higher order aggregates are rare. Aggregates are formed more rarely in simulations containing pleurocidin-KR. Aggregates are more likely in simulations containing WF1a alone or mixed with WF2 but even in this case there are two simulations where practically no aggregates are formed at all.

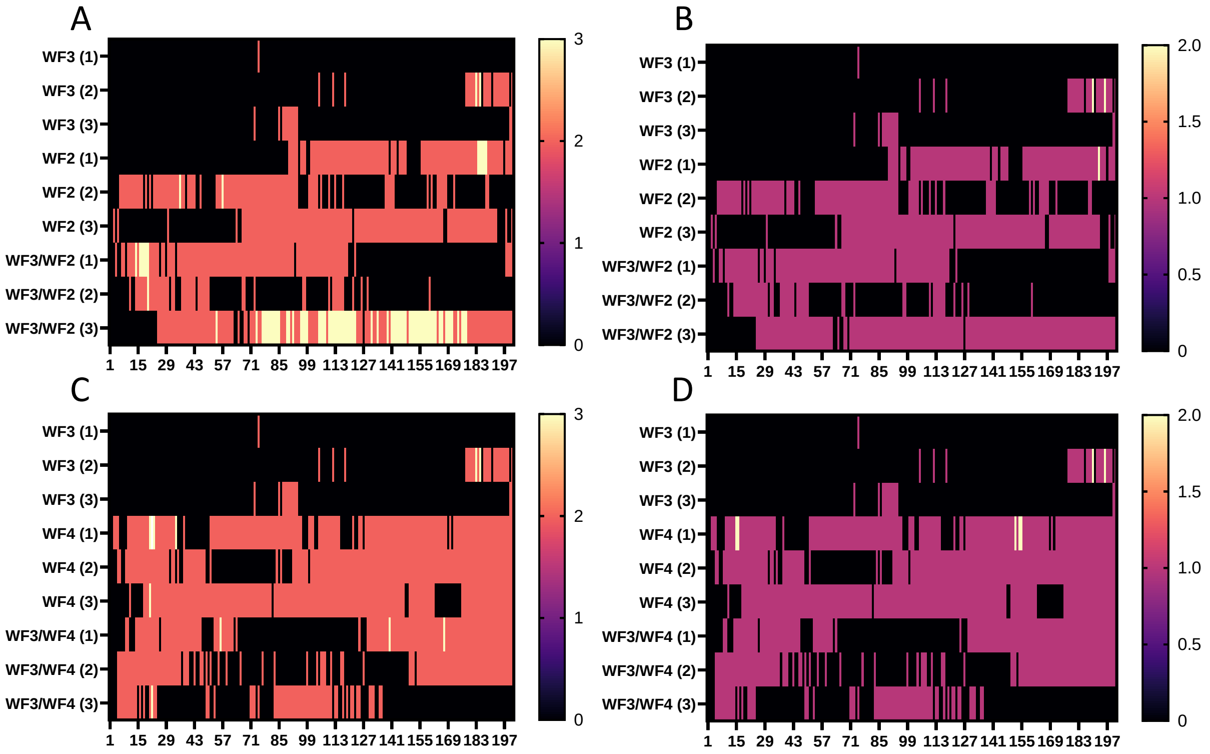

Supplementary Figure 8. Aggregation analyses for WF AMPs in the MD simulations. The number of peptides that cluster together at any one time (A, C) and the number of such clusters at any one time (B, D) are shown as a function of time over the duration of the 200 ns simulation for the indicated peptides when binding and inserting into POPE/POPG bilayers. There are four WF AMPs in each simulation – either four WF2, WF3 or WF4 when unmixed, or for the combinations two WF3 plus either two WF2 or two WF4. Dimers are formed frequently but higher order aggregates are rare. Aggregates are formed more rarely in simulations containing WF3. Aggregates are more likely in simulations containing WF4 alone.

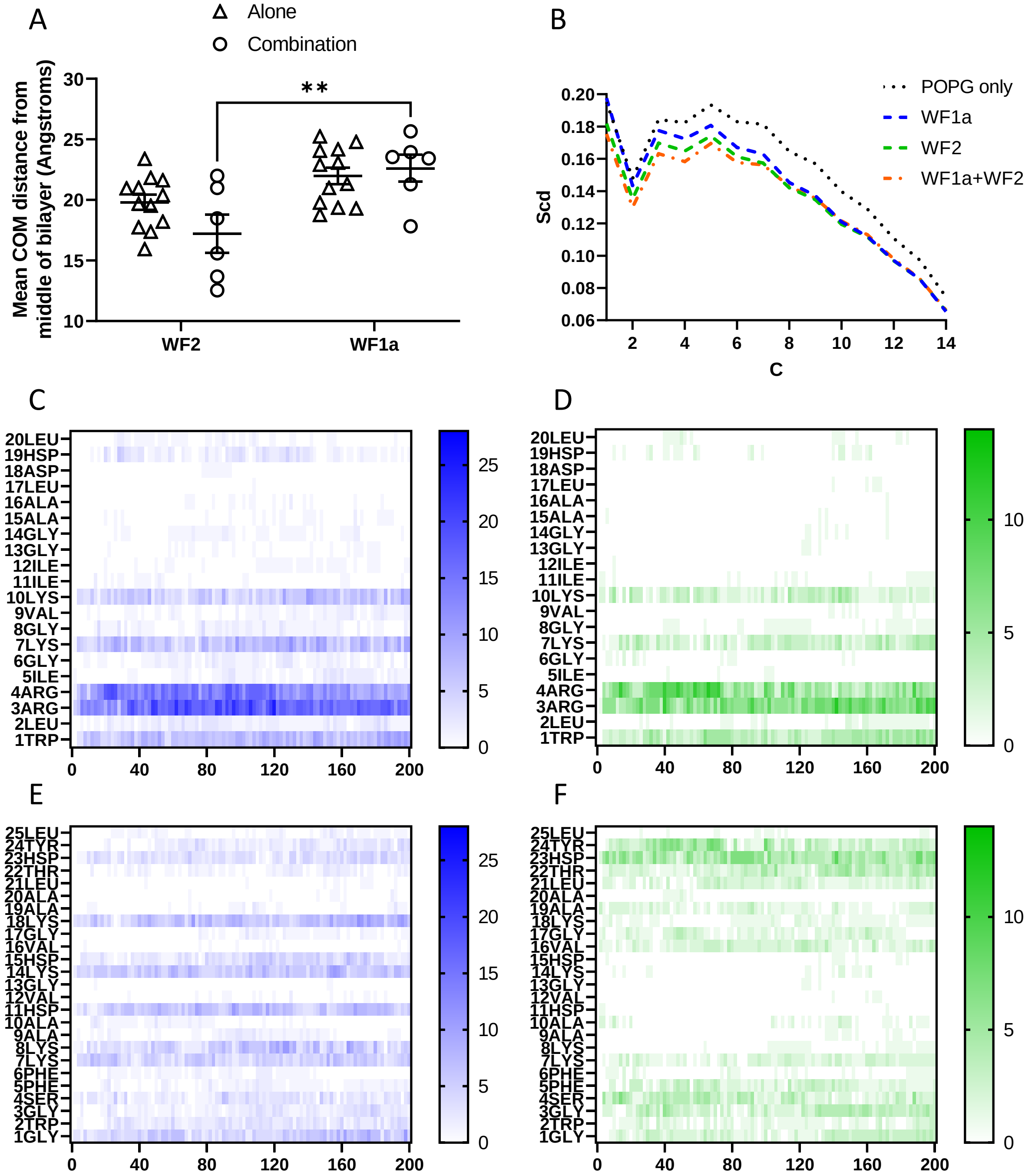

Supplementary Figure 9. MD simulation – altered penetration and H-bonding distribution in synergistic combinations of WF2 and WF1a inserting into POPG bilayers. Centre of mass analysis where each point is one peptide in one of three replicate simulations, the bar is the mean and error SEM (A). Lipid acyl chain order parameters for lipids within 4 Å of a peptide shown as averages of the three replicates (B). Hydrogen bonding distribution for WF1a (C, D) or WF2 (E, F) run unmixed (C, E) or as combinations (D, F). Each panel is a sum of four peptides when the peptides are unmixed or two peptides each for the combinations and is representative of n = 3 replicates.

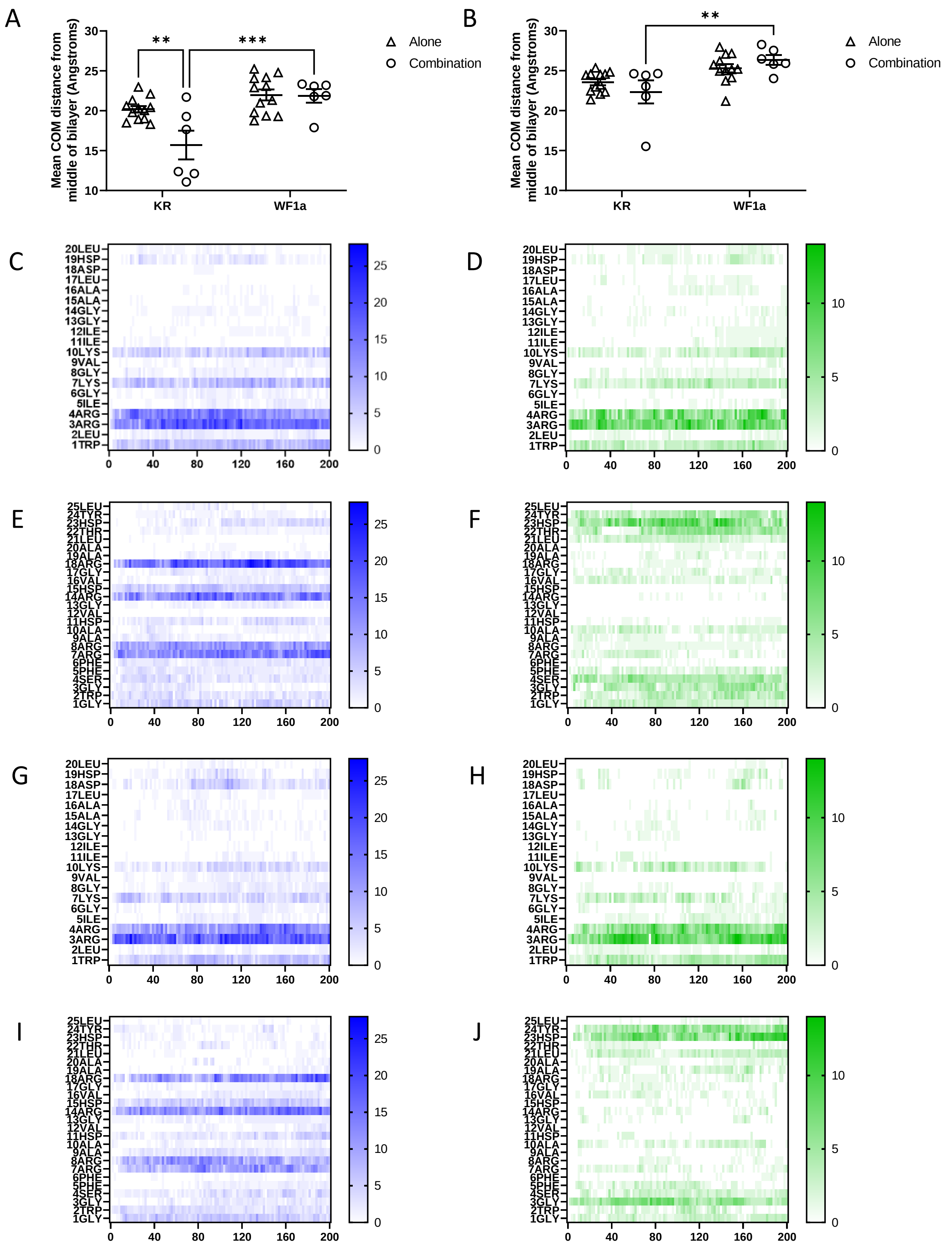

Supplementary Figure 10. MD simulation – altered H-bonding distribution in synergistic combinations of WF1a and pleurocidin-KR inserting into POPG or POPE/POPG bilayers. Centre of mass analysis for POPG (A) and POPE/POPG (B) where each point is one peptide in one of three replicate simulationsthe bar is the mean and error SEM. Hydrogen bonding distribution for WF1a (C, D, G, H) or pleurocidin-KR (E, F, I, J) run unmixed (C, C, G, I) or as combinations (D, F, H, J). Each panel is a sum of four peptides when the peptides are unmixed or two peptides each for the combinations and is representative of n = 3 replicates. Note for POPE/POPG, but not POPG, bilayers total intermolecular H-bonding increases for pleurocidin KR (p = 0.0373) when in combination with WF1a.

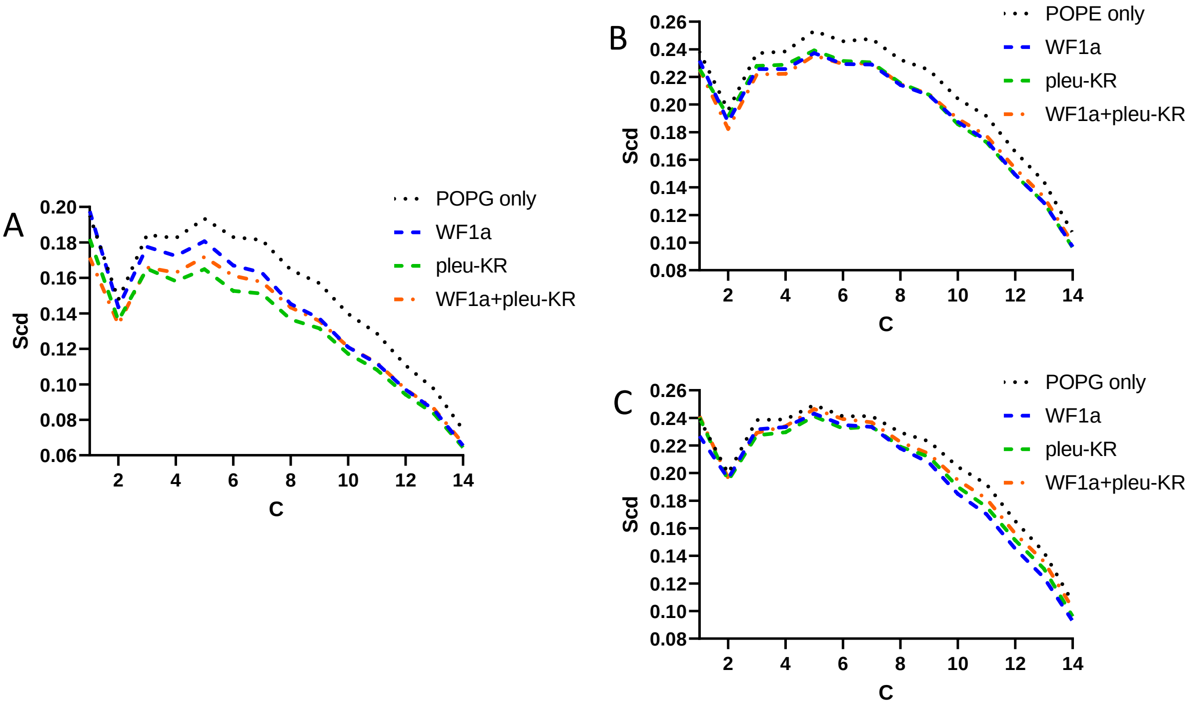

Supplementary Figure 11. MD simulations – disordering of lipids by WF1a, pleurocidin-KR and their combination. Lipid acyl chain order parameters for lipids within 4 Å of a peptide shown as averages of the three replicates for POPG bilayers (A) and POPE (B) and POPG (C) in POPE/POPG bilayers.

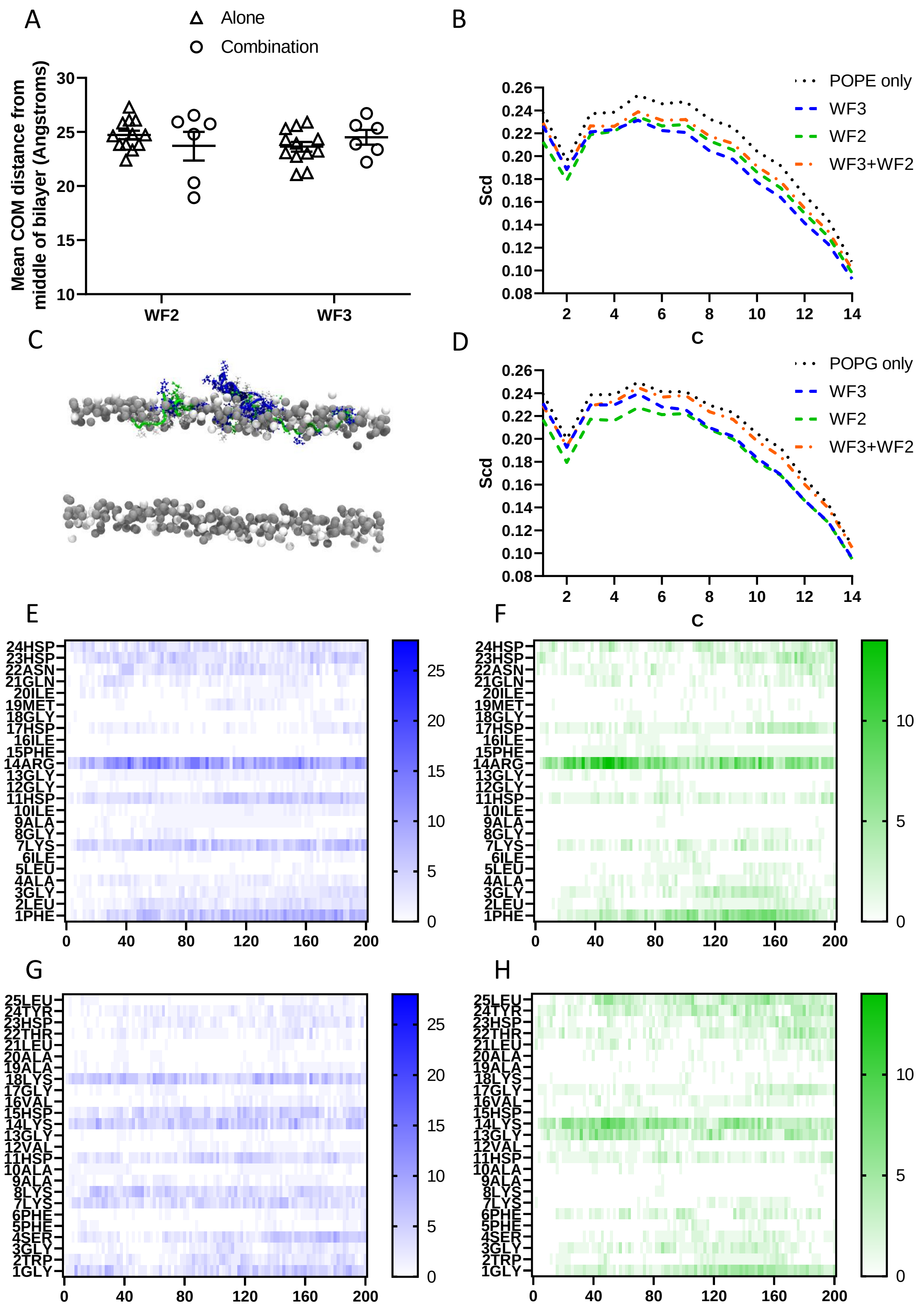

Supplementary Figure 12. MD simulation – altered H-bonding distribution in synergistic combinations of WF3 and WF2 inserting into POPE/POPG bilayers. Centre of mass analysis where each point is one peptide in one of three replicate simulations, the bar is the mean and error SEM (A), with a representative snapshot at 200 ns (C; WF3 green). Lipid acyl chain order parameters for lipids within 4 Å of a peptide shown as averages of the three replicates for POPE (B) or POPG (D). Hydrogen bonding distribution for WF3 (E, F) or WF2 (G, H) run unmixed (E, G) or as combinations (F, H). Each panel is a sum of four peptides when the peptides are unmixed or two peptides each for the combinations and is representative of n = 3 replicates. There is no change in total number of intermolecular H-bonding for either peptide between unmixed and combination.

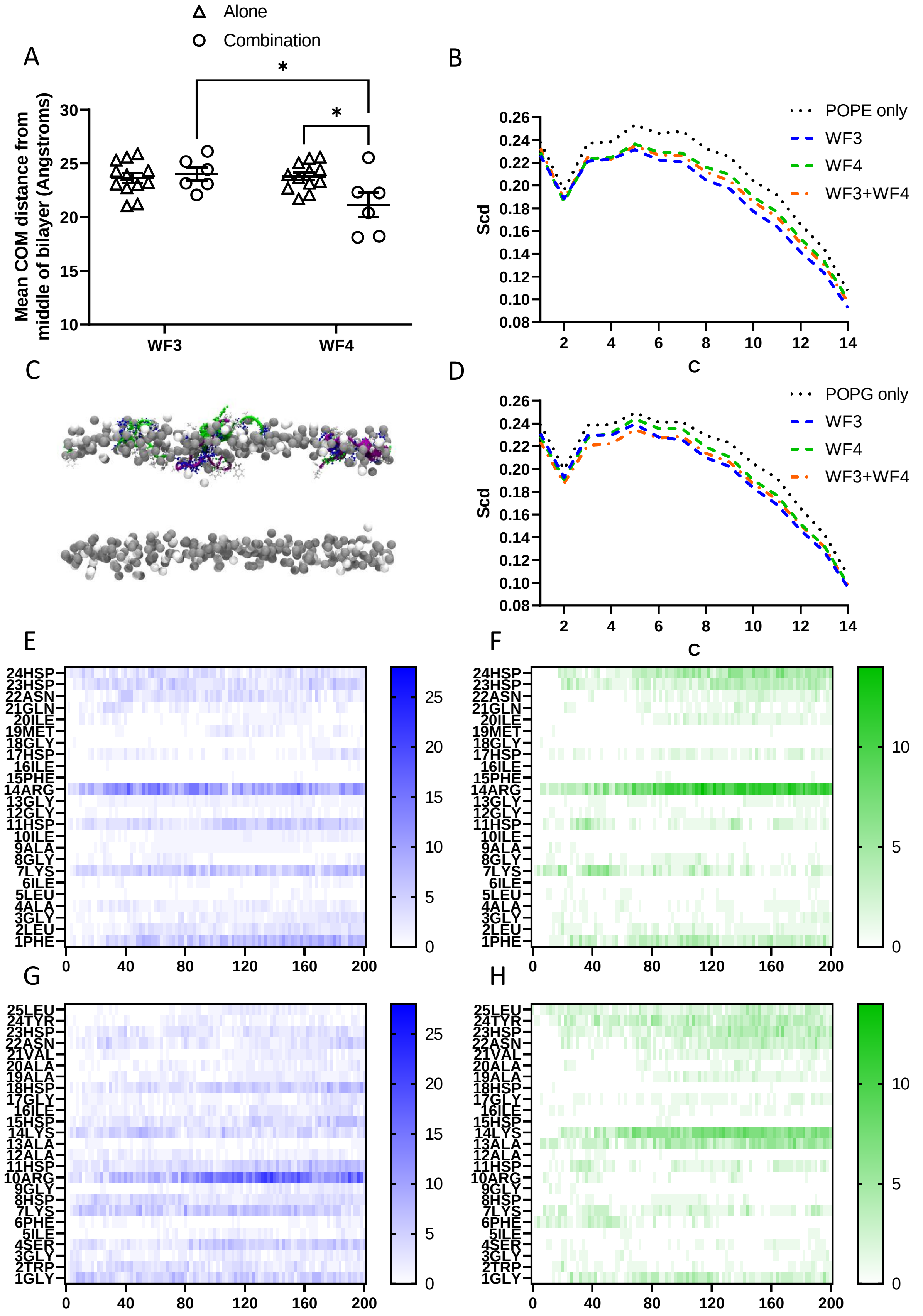

Supplementary Figure 13. MD simulation – altered H-bonding distribution in synergistic combinations of WF3 and WF4 inserting into POPE/POPG bilayers. Centre of mass analysis where each point is one peptide in one of three replicate simulationsthe, bar is the mean and error SEM (A), and a representative snapshot at 200 ns (C; WF4 purple). Lipid acyl chain order parameters for lipids within 4 Å of a peptide shown as averages of the three replicates for POPE (B) or POPG (D). Hydrogen bonding distribution for WF3 (E, F) or WF4 (G, D) run unmixed (E, G) or as combinations (F, H). Each panel is a sum of four peptides when the peptides are unmixed or two peptides each for the combinations and is representative of n = 3 replicates. There is no change in total number of intermolecular H-bonding for either peptide between unmixed and combination.

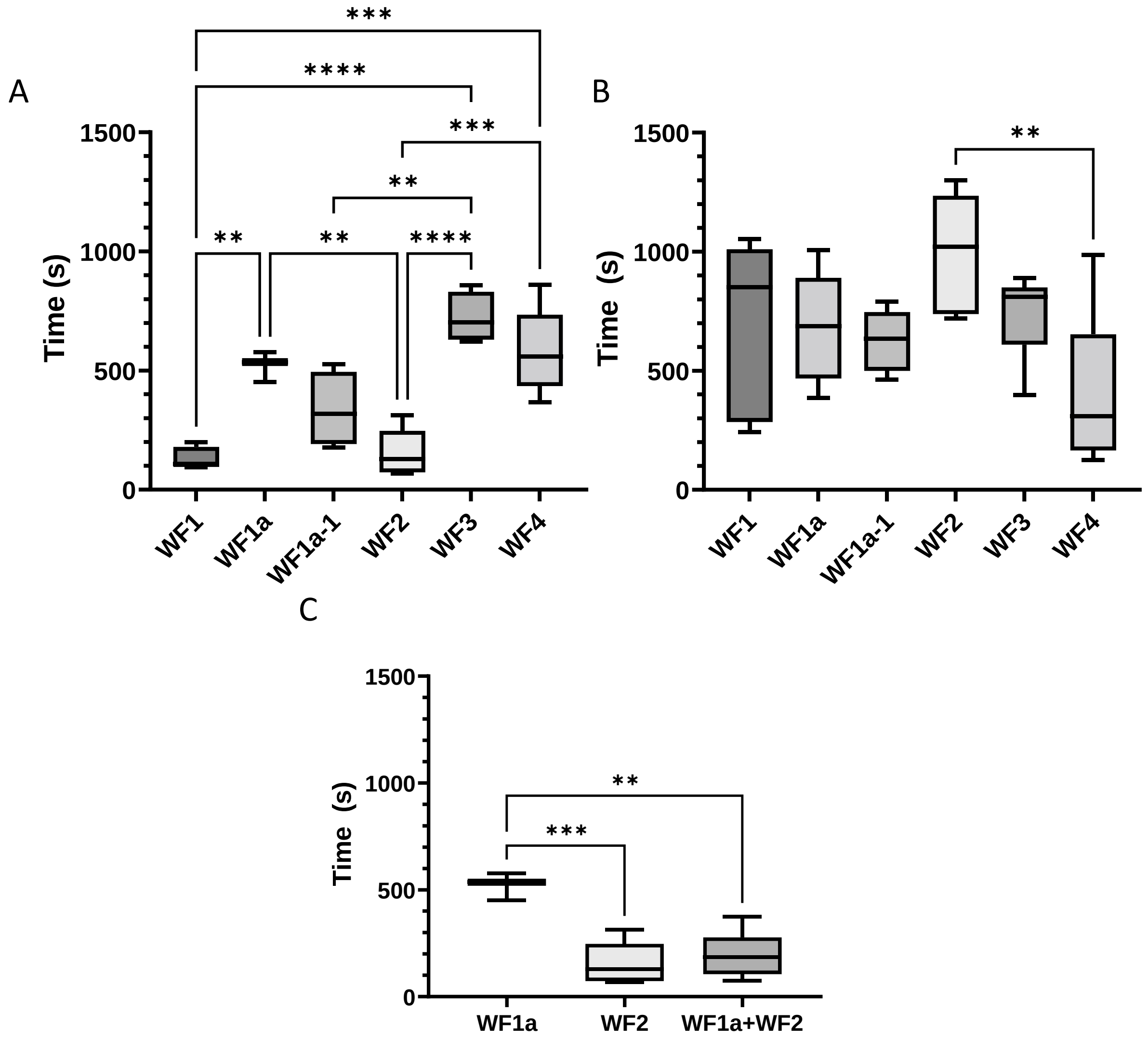

Supplementary Figure 14. Combining WF1a and WF2 ensures rapid onset of ion conductance. Latency – time taken for detectable conductance to be recorded after addition of peptide at threshold concentration – is shown for all six WF peptides in DPhPG (A) or DPhPE/DPhPG (B) and the combination of WF1a and WF2 in DPhPG (C). For the model of the Gram-positive plasma membrane the combination matches the performance of WF2 alone. For the Gram-negative model the combination ensures that ion conductance that could be associated with a bactericidal effect occurs more rapidly. In each case a one-way ANOVA with Tukey's multiple comparison test is performed ($p < 0.05$ *; $p < 0.01$ **; $p < 0.001$ ***; $p < 0.0001$ ****). Boxes are 25th to 75th percentiles, whiskers are minimum to maximum value and the centre line indicates the median.
