# Supplementary material for: Synergy between Winter Flounder antimicrobial peptides
Source: NPJ Antimicrob Resist. 2023 Aug 10;1:8. doi: 10.1038/s44259-023-00010-7 (PMC11057203; doi:10.1038/s44259-023-00010-7)
Supplement: Supplementary file 2 — REPORTING SUMMARY [file 44259_2023_10_MOESM2_ESM.pdf]

nature portfolio

Corresponding author(s): Prof Andrew Mason

Last updated by author(s): Jun 2, 2023

Reporting Summary

Nature Portfolio wishes to improve the reproducibility of the work that we publish. This form provides structure for consistency and transparency in reporting. For further information on Nature Portfolio policies, see our Editorial Policies and the Editorial Policy Checklist.

Statistics

For all statistical analyses, confirm that the following items are present in the figure legend, table legend, main text, or Methods section.

n/a	Confirmed	
☐	☒	The exact sample size (*n*) for each experimental group/condition, given as a discrete number and unit of measurement
☐	☒	A statement on whether measurements were taken from distinct samples or whether the same sample was measured repeatedly
☐	☒	The statistical test(s) used AND whether they are one- or two-sided<br>*Only common tests should be described solely by name; describe more complex techniques in the Methods section.*
☒	☐	A description of all covariates tested
☒	☐	A description of any assumptions or corrections, such as tests of normality and adjustment for multiple comparisons
☒	☐	A full description of the statistical parameters including central tendency (e.g. means) or other basic estimates (e.g. regression coefficient) AND variation (e.g. standard deviation) or associated estimates of uncertainty (e.g. confidence intervals)
☒	☐	For null hypothesis testing, the test statistic (e.g. *F*, *t*, *r*) with confidence intervals, effect sizes, degrees of freedom and *P* value noted<br>*Give P values as exact values whenever suitable.*
☒	☐	For Bayesian analysis, information on the choice of priors and Markov chain Monte Carlo settings
☒	☐	For hierarchical and complex designs, identification of the appropriate level for tests and full reporting of outcomes
☒	☐	Estimates of effect sizes (e.g. Cohen's *d*, Pearson's *r*), indicating how they were calculated

Our web collection on statistics for biologists contains articles on many of the points above.

Software and code

Policy information about availability of computer code

Data collection	MD simulations were done using Gromacs 2018 or 2020 using the CHARMM36 forcefield.
Data analysis	In vitro PD, patch-clamp traces and CD data were analysed with OriginPro 2020, NMR structure calculations were performed with Dynamo. All statistical comparisons were done in GraphPad Prism 9.

For manuscripts utilizing custom algorithms or software that are central to the research but not yet described in published literature, software must be made available to editors and reviewers. We strongly encourage code deposition in a community repository (e.g. GitHub). See the Nature Portfolio guidelines for submitting code & software for further information.

Data

Policy information about availability of data

All manuscripts must include a data availability statement. This statement should provide the following information, where applicable:

- Accession codes, unique identifiers, or web links for publicly available datasets
- A description of any restrictions on data availability
- For clinical datasets or third party data, please ensure that the statement adheres to our policy

Supplementary Information including more extensive analysis of the MD simulation data, Circular Dichroism experiments and further analysis of the patch-clamp data. Structural coordinates were deposited in the Protein Data Bank (www.rcsb.org) and Biological Magnetic Resonance Bank (BMRB; www.bmrb.wisc.edu) under acces-sion codes of 6S2D, 6RYQ, 6RY9, 6RZ1 and 6RZC (PDB) and 34416, 34411, 34410, 34412, and 34413 (BMRB) for WF1, WF1a, WF1a-1, WF3 and WF4

respectively. In addition to the structural coordinates the datasets gen-erated during and/or analysed during the current study are available from the corresponding author on reason-able request.

Research involving human participants, their data, or biological material

Policy information about studies with human participants or human data. See also policy information about sex, gender (identity/presentation), and sexual orientation and race, ethnicity and racism.

Reporting on sex and gender	The research does not use human participants.
Reporting on race, ethnicity, or other socially relevant groupings	The research does not use human participants.
Population characteristics	The research does not use human participants.
Recruitment	The research does not use human participants.
Ethics oversight	The research does not use human participants.

Note that full information on the approval of the study protocol must also be provided in the manuscript.

Field-specific reporting

Please select the one below that is the best fit for your research. If you are not sure, read the appropriate sections before making your selection.

☒ Life sciences ☐ Behavioural & social sciences ☐ Ecological, evolutionary & environmental sciences

For a reference copy of the document with all sections, see nature.com/documents/nr-reporting-summary-flat.pdf

Life sciences study design

All studies must disclose on these points even when the disclosure is negative.

Sample size	This is relevant only to the Galleria mellonella studies where a minimum of three repeats of n = 10 caterpillars per group is necessary to register significant effects with a four day monitoring period, assuming independent repeats are consistent. The sample size is limited by the number of treatment groups that an operator can reasonably set up in each session.
Data exclusions	No data was excluded.
Replication	With the exception of NMR structural studies, all experiments were independently replicated three times.
Randomization	Treatments groups for Galleria mellonella experiments were
Blinding	Blinding is not relevant for any of the studies here. For Galleria mellonella experiments the outcomes are binary and none of the data produced from the other methods is subjective.

Reporting for specific materials, systems and methods

We require information from authors about some types of materials, experimental systems and methods used in many studies. Here, indicate whether each material, system or method listed is relevant to your study. If you are not sure if a list item applies to your research, read the appropriate section before selecting a response.

Materials & experimental systems

n/a	Involved in the study	
☒	☐	Antibodies
☒	☐	Eukaryotic cell lines
☒	☐	Palaeontology and archaeology
☐	☒	Animals and other organisms
☒	☐	Clinical data
☒	☐	Dual use research of concern
☒	☐	Plants

Methods

n/a	Involved in the study	
☒	☐	ChIP-seq
☒	☐	Flow cytometry
☒	☐	MRI-based neuroimaging

Animals and other research organisms

Policy information about studies involving animals; ARRIVE guidelines recommended for reporting animal research, and Sex and Gender in Research

Laboratory animals	The study involved only Galleria mellonella for which ethical approval is not required.
Wild animals	The study did not involve wild animals.
Reporting on sex	Not applicable.
Field-collected samples	The study did not involve samples collected from the field.
Ethics oversight	No ethical approval was required as there are no organisms involved in the study whose use is controlled by Home Office or other ethical committees.

Note that full information on the approval of the study protocol must also be provided in the manuscript.
